# Mathematical Model of the Firefly Luciferase Complementation Assay Reveals a Non-Linear Relationship between the Detected Luminescence and the Affinity of the Protein Pair Being Analyzed

**DOI:** 10.1371/journal.pone.0148256

**Published:** 2016-02-17

**Authors:** Renee Dale, Yuki Ohmuro-Matsuyama, Hiroshi Ueda, Naohiro Kato

**Affiliations:** 1 Department of Biological Sciences, Louisiana State University, Baton Rouge, Louisiana, United States of America; 2 Chemical Resources Laboratory, Tokyo Institute of Technology, Nagatsuta-cho, Yokohama, Kanagawa, Japan; National Institutes of Health, UNITED STATES

## Abstract

The firefly luciferase complementation assay is widely used as a bioluminescent reporter technology to detect protein-protein interactions *in vitro*, *in cellulo*, and *in vivo*. Upon the interaction of a protein pair, complemented firefly luciferase emits light through the adenylation and oxidation of its substrate, luciferin. Although it has been suggested that kinetics of light production in the firefly luciferase complementation assay is different from that in full length luciferase, the mechanism behind this is still not understood. To quantitatively understand the different kinetics and how changes in affinity of a protein pair affect the light emission in the assay, a mathematical model of the *in vitro* firefly luciferase complementation assay was constructed. Analysis of the model finds that the change in kinetics is caused by rapid dissociation of the protein pair, low adenylation rate of luciferin, and increased affinity of adenylated luciferin to the enzyme. The model suggests that the affinity of the protein pair has an exponential relationship with the light detected in the assay. This relationship causes the change of affinity in a protein pair to be underestimated. This study underlines the importance of understanding the molecular mechanism of the firefly luciferase complementation assay in order to analyze protein pair affinities quantitatively.

## Introduction

The firefly luciferase complementation assay (FLCA) is an assay that detects protein-protein interactions *in vitro*, *in cellulo*, and *in vivo* [1, 2]. The assay detects the bioluminescence that is emitted during the oxidation of the substrate, D-luciferin (LH_2_). In the assay, the N-terminal and C-terminal domain of firefly luciferase (NFLuc and CFLuc, respectively) are genetically fused to a protein pair of interest via a linker peptide [3]. When the protein pair interacts with each other, NFLuc and CFLuc reconstitute the active site of the enzyme. This results in emission of luminescence when LH_2_ is added in the reaction.

The FLCA can be used in many different organisms and systems. In the *in vitro* assays previously conducted [4], the substrates LH_2_ and ATP are added to a 96-well plate containing a buffer and a protein pair fused to NFLuc and CFLuc, respectively. Luminescence is measured in relative units (RLU) with a photomultiplier tube. In the *in cellulo* assay, the cells of interest are suspended in a culture plate. LH_2_ is added to the culture plate so that the substrate contacts with the enzyme *via* diffusion through the cell membranes. When performing FLCA *in vivo*, LH_2_ can be injected into the circulatory system [5]. The typical FLCA luminescence kinetics include a delayed peak followed by slow decay [4, 6]. The highest RLU detected during the assay is generally used to evaluate the interaction of the protein pair.

The molecular mechanism of full length firefly luciferase reaction has been well established. During the reaction, the substrate LH_2_ is adenylated to form the intermediate luciferyl-adenylate (LH_2_-AMP) (reaction 1). LH_2_-AMP is oxidized to from excited oxyluciferin (L-oxy⋆, reaction 2) which emits light when it decays to its ground state (L-oxy, reaction 3) [7]. The dark reaction product dehydroluciferin-AMP (L-AMP, reaction 4) is formed in an alternate chemical pathway [7]. The dark reactions account for approximately 20% of all luciferase activity [7]. Both of these products, L-oxy and L-AMP, inhibit luciferase competitively against LH_2_[6]. Firefly luciferase is a 62 kDa peptide encoded by 550 amino acids [8]. X-ray crystallography has revealed that the N domain is encoded in amino acids 4–436, and the C domain in amino acids 440–544 [8]. There is a flexible hinge region between the two domains at amino acids 436–440 [8]. The flexible hinge region allows the C domain to change conformation during the oxidation step, allowing the catalytic residue to come in contact with the substrate. Both of the primary amino acids involved in catalysis are found on the C domain. Amino acid K529 is responsible for adenylation of the substrate, while K443 is responsible for oxidation of the intermediate [9, 10]. Gene mutation studies have identified amino acids 213–348 of the N domain as binding sites for LH_2_ and ATP [11]. N domain residue H245 is considered the key binding residue, as it is highly conserved throughout the acetyl-CoA synthetase, non-ribosomal protein synthetase, and luciferase (ANL) superfamily in addition to being in the region identified as responsible for binding of substrates [11].

$$\text{Luc}+LH_{2}+ATP\;\overset{Mg^{2+}}{⇄}\;\text{Luc}\cdot LH_{2}-AMP+PP_{i}$$ {1}

$$\begin{array}{c} \text{Luc}\;\cdot LH_{2}-AMP+O_{2}\;\to \;\text{Luc}\;\cdot L-oxy⋆+AMP+CO_{2} \end{array}$$ {2}

$$\begin{array}{c} \text{Luc}\;\cdot L-oxy⋆\;\to \;\text{Luc}\;\cdot \;L-oxy+hv \end{array}$$ {3}

$$\begin{array}{c} \text{Luc}\;\cdot LH_{2}-AMP+O_{2}\to \text{Luc}\;\cdot L-AMP+H_{2}O_{2} \end{array}$$ {4}

**Fig 1:**
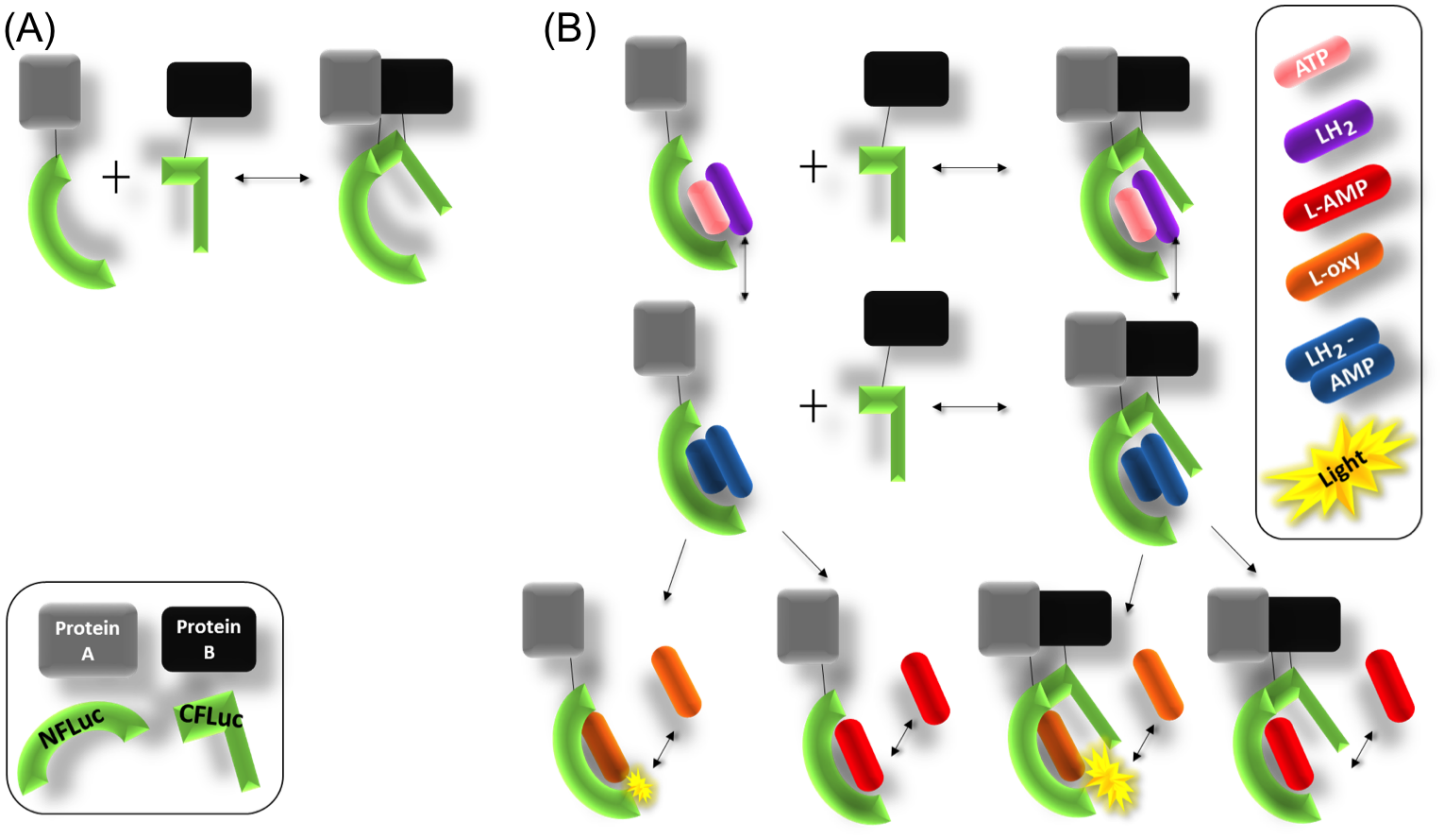
Overview of *in vitro* firefly Luciferase complementation assay (FLCA) system. (A) With interaction of a protein pair (shown here, protein A and B), the N and C domains of luciferase (NFLuc and CFLuc, respectively) reconstitute the active site of the enzyme. The amount of the reconstituted enzyme (NC complex) is thought to correlate with the affinities of the protein pair. (B) Upon the addition of the substrates, LH_2_ and ATP, catalysis occurs in a two step process. The enzyme first adenylate LH_2_ with ATP, forming the intermediate LH_2_-AMP. The intermediate is then oxidized to form L-oxyluciferin (L-oxy) during the light emission reaction. Alternatively, the intermediate is oxidized to form dehydroluciferyl-AMP (L-AMP) without emitting light (dark reaction). Both products inhibit luciferase activity competitively. NFLuc has low luciferase-activity on its own [12, 13].

In the FLCA, NFLuc consists of amino acids 1–437, and CFLuc contains 395–547 [4]. The overlapping region common to both NFLuc and CFLuc, amino acids 395–437, includes the flexible hinge region and part of the N domain. Although the FLCA is valued for its simple protocol, it is involved in a complex system of enzymatic reactions (Fig 1). While NFLuc alone has residual enzymatic activity (binding and catalysis of the substrates), CFLuc is key in increasing the efficiency of catalysis [9, 10, 12, 13]. Hence in the FLCA, the affinity of a protein pair fused to NFLuc or CFLuc via a linker peptide influences the luminescence output by altering the interaction between NFLuc and CFLuc.

Previously published FLCA data has interpreted protein pair interactions qualitatively (the presence or absence of protein interactions) or quantitatively (affinities of protein interactions are compared based on the RLU) [1, 2, 14–16]. We searched for articles with the keywords ‘luciferase complementation assay’ using Google Scholar, and identified the first 46 peer-reviewed articles in the result list. Among the 46 articles, we found that 50% of 46 previously published peer-reviewed articles using the FLCA claim that the maximum RLU detected during the assay is a quantitative measurement of the affinity of the protein interactions [5, 14, 17–60]. In such articles, FLCA data are considered quantitative because of an assumption that the reconstituted activity of NFLuc and CFLuc is entirely and linearly dependent upon the affinity of the protein pair of interest and the concentration of the interacting complex.

However, the relationship between changes of the affinity of the protein pair and the luminescence detected with the FLCA has not been quantitatively understood. In fact, it has been previously suggested that the kinetics of luminescence production in the FLCA is different from that in full length firefly luciferase [12–14]. Without a thorough understanding of the cause for these changes in the luminescence kinetics, or a demonstration of the relationship between protein interaction and luminescence, the FLCA cannot reasonably be considered a quantitative measure of either protein pair affinity or the concentration of the protein pair complex. The purpose of this study is to quantitatively understand the relationship between changes of the affinity of a protein pair and the luminescence detected in the FLCA using a mathematical model. The model is constructed with ordinary differential equations (ODEs) using known enzymatic reactions and equilibrium constants for both firefly luciferase and the protein pair. Using the model, we analyze the degree to which luminesence observed in the FLCA is affected by the interaction of a protein pair.

## Materials and Methods

### Measurement of the kinetics of *in vitro* FLCA

Kinetic data of luminescence production in FLCA was obtained under the same conditions as previously published in [4]. Briefly, purified recombinant protein of p53 and mdm2 fused to NFLuc and CFLuc (50 nM each) were suspended in a 2x enzyme solution containing 100 mM MOPS, 10 mM MgSO_4_, pH 7.3 [4]. The mixture (50 *μ*l) was dispensed to a well in a white 96-well plate (Corning-Costar, NY, USA) after incubation at 37°C for 120 s. The light intensity was measured immediately after injection of 50 *μ*l 2x substrate solution (40 mM ATP and 150 *μ*M LH_2_ in 100 mM MOPS, 10 mM MgSO_4_, pH 7.3) with a periodical integration for 0.1 s using Phelios AB-2350 luminometer (ATTO, Tokyo, Japan).

### Measurement of the kinetics of *in vitro* full length firefly luciferase

Kinetic data of luminescence production in full length firefly luciferase was obtained under the same conditions as that in FLCA. Briefly, firefly luciferase was purchased from Promega (Wisconsin, USA). Luciferase enzyme (150 nM or 450 nM) was suspended in 100 mM MOPS, 19 mM MgSO_4_, pH 7.3. One microliter of the enzyme solution and 50 *μ*L of a 2x ATP solution (40 mM ATP in 100 mM MOPS, 10 mM MgSO_4_, pH 7.3) was dispensed to a well in a white 96-well plate (Corning-Costar, NY, USA). The luminescence was measured immediately after injection of a 2x LH_2_ solution (150 *μ*M LH_2_, 100 mM MOPS, 10 mM MgSO_4_, pH 7.3) with periodical integration for 0.2 s for 120 s using Synergy 2 luminometer (Biotek, Vermont, USA).

### Calculation of the degradation of NFLuc and CFLuc

The degradation of NFLuc and CFLuc were previously analyzed and published [4]. To obtain a degradation rate for these species, the data were analyzed using Eq 1 which describes exponential degradation. The maximum RLU, representative of relative enzymatic activity, was digitized for each incubation time using PlotDigitizer and normalized [61]. The degradation rate was calculated by curve fitting to Eq 1 using Matlab’s nlinfit function for nonlinear regression [62].

$$\begin{array}{c} Activity=e^{-DegradationRate\cdot Time} \end{array}$$ (1)

### Estimation of parameters

Initial estimates for parameters were taken from the literature. Initial estimates for all k_on_ rates were held between the physiologically relevant range of 10^5^−10^8^ *M*^−1^ throughout the optimization process [63]. Parameters unavailable from the literature were estimated by curve fitting the model to previous data. Ordinary differential equations (ODEs) were numerically solved with MatLab’s ode23s for stiff systems [62]. The curve fitting was done using MatLab’s lsqcurvefit function.

### Calculation of initial conditions

The initial concentrations of the non-interacting and interacting protein pair prior to the addition of substrates was calculated with the system of equations shown in S1 ODE. This uses the *k*_*on*_ and *k*_*off*_ for the protein pair, the initial concentration of the proteins, and the degradation rate of NFLuc and CFLuc. The *k*_*on*_ and *k*_*off*_ of the protein pair, here p53 and mdm2, was obtained from the literature [64]. The incubation time was estimated for 1 s due to the delay associated with manual portions of the procedure when no incubation time was defined by the experimenters.

### Calculation of the IC-50

The K_i_ of nutlin-3 was estimated using Eq 2 [65]. To calculate the IC-50 from the model simulation of the p53 and mdm2 interaction, we first plotted the simulated RLU at 0.2 s, following the previous experimental procedure [4]. We then fit these points to Eq 3 using nonlinear regression. This equation is a 4 parameter logistic model, where the parameters min and max are asymptotes which the data approaches but does not touch. The IC-50 point is a calculated number halfway between these two asymptotes. Nonlinear regression and plotting was accomplished using a modified form of the independently designed DoseResponse package for Matlab [66].

$$\begin{array}{c} K_{i}=\frac{IC_{50}}{\frac{\left[NFLuc\right]}{K_{d}}+1} \end{array}$$ (2)

$$\begin{array}{c} Activity=max+\frac{min-max}{1+\frac{\left[nutlin-3\right]}{IC-50}} \end{array}$$ (3)

### Simulation of full length kinetics

The mathematical model describing the FLCA was stripped down to simulate only the NC complex (S4 ODE). In order to calculate the k_off_s for full length luciferase, we used the k_on_s obtained from the optimization of the NC complex and dissociation constants from the literature, using Eq 4 (S2 Table).

$$\begin{array}{c} K_{d}=\frac{k_{off}}{k_{on}} \end{array}$$ (4)

## Results and discussion

### Kinetics of light production in the FLCA and full length luciferase are different

**Fig 2:**
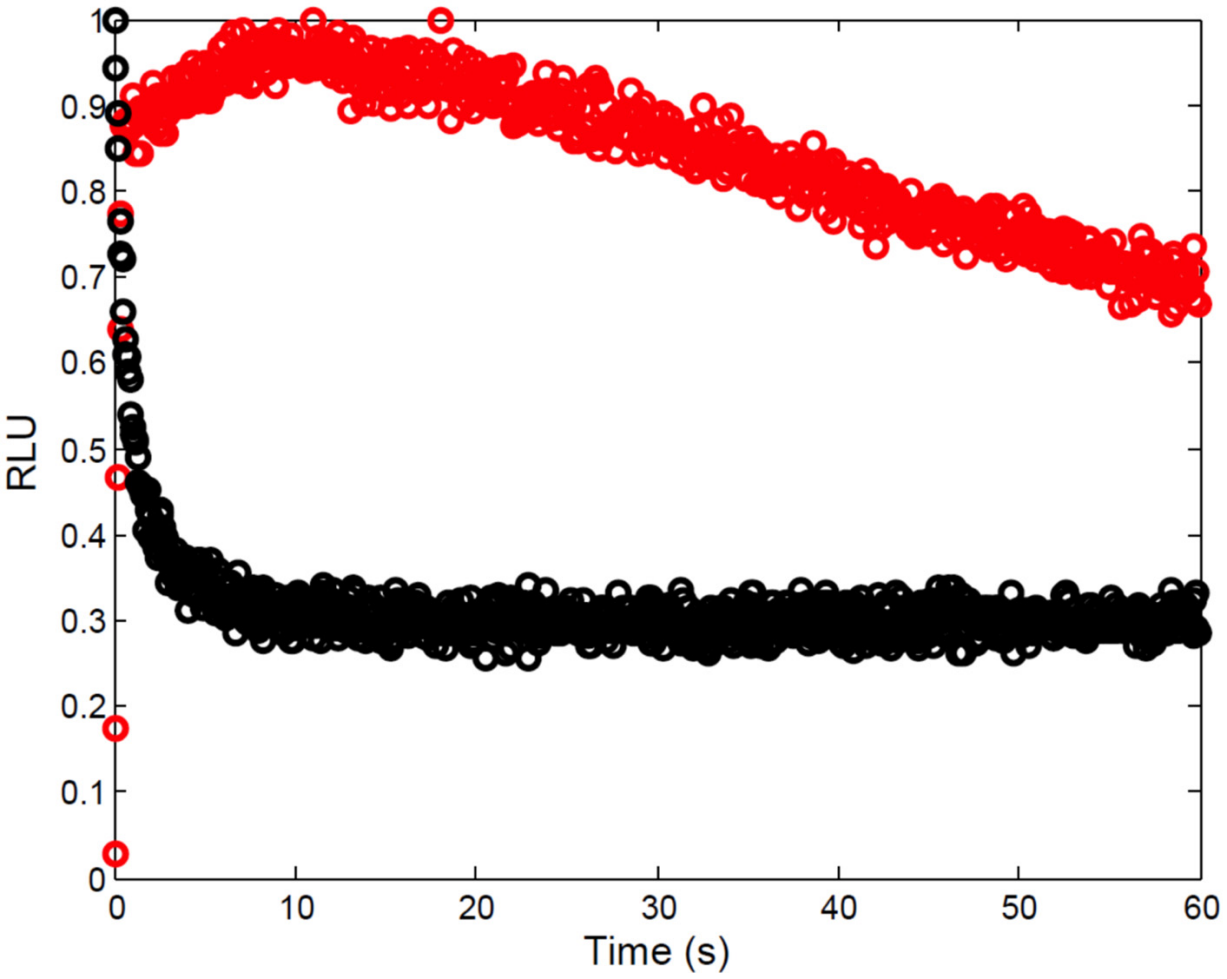
Luminescence kinetics of full length luciferase (black) and of the FLCA (red) are different. Changes in the relative luminescence of full length luciferase and of the FLCA *in vitro* were monitored every 0.2 s and 0.1 s, respectively, for 120 s in a 96-well plates. Detected luminescence was normalized so that the maximum luminescence in each assay is 1. Notice that the kinetics of 150 nM full length firefly luciferase has a sharp peak within 1 s followed by quick decay. On the other hand, the kinetics of 50 nM NFLuc and 50 nM CFLuc has a more delayed peak and slower decay.

Previous independent studies suggest that the kinetics of luminescence production in full length luciferase and those of the FLCA would be different [12–14]. We therefore conducted experiments that directly compared the kinetics of luminescence production in the FLCA and full length luciferase (Fig 2). In this experiment, we used 50 nM each of p53-NFLuc and mdm2-CFLuc. The proteins p53 and mdm2 are known to interact with each other *in vitro* with a dissociation constant (K_d_) of 212 nM [64].

In our experimental conditions, the luminescence kinetics of 150 nM full length firefly luciferase was measured after adding 75 *μ*M LH_2_ and 100 mM ATP. Full length firefly luciferase shows a sharp peak within the first second, followed by fast signal decay (Fig 2). These kinetics are observed regardless of the different concentrations of full length firefly luciferase used (S1 Fig) [7]. On the other hand, the luminescence kinetics of the FLCA shows a slower peak with slower signal decay (Fig 2). Previous data shows that the luminescence kinetics of FLCA is independent from protein concentrations used in the assays [4]. Our results, therefore, confirmed the previous suggestion that the kinetics of luminescence production in the FLCA and full length luciferase are different. This result also suggested that the different luminescence kinetics are due to factors other than insufficient substrates in the reaction solution.

### Functions of the N and C domains in firefly luciferase were incorporated into a mathematical model

**Fig 3:**
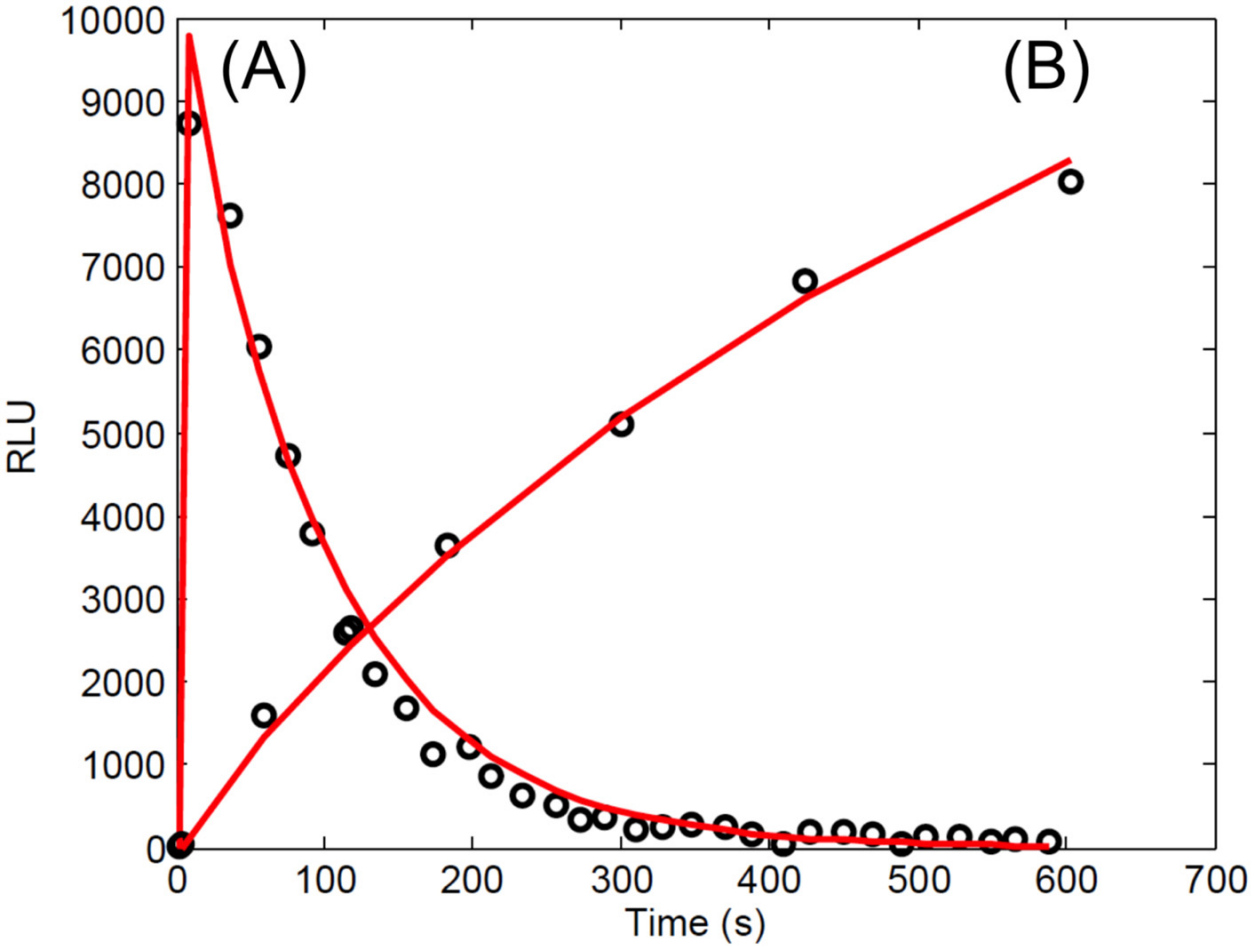
Curve fit of the *in vitro* luminesence kinetics of the N domain of firefly luciferase. Data originally published in [12] was digitized using Plot Digitizer [61]. Digitized data was curve fit to estimate parameters unavailable from previously published papers. (A) The addition of 3.7 nM LH_2_-AMP to 1 *μ*M of the N domain shows a sharp peak. This curve fit provided an estimation of the adenylation forward and reverse rates. (B) When a substrate solution (300 *μ*M LH_2_, 10 mM ATP) is added to 1 *μ*M of the N domain, the luminescence kinetics have a slow rise and no peak. This curve fit provided more optimized values for the available NFLuc alone binding and catalysis rates in the FLCA.

**Fig 4:**
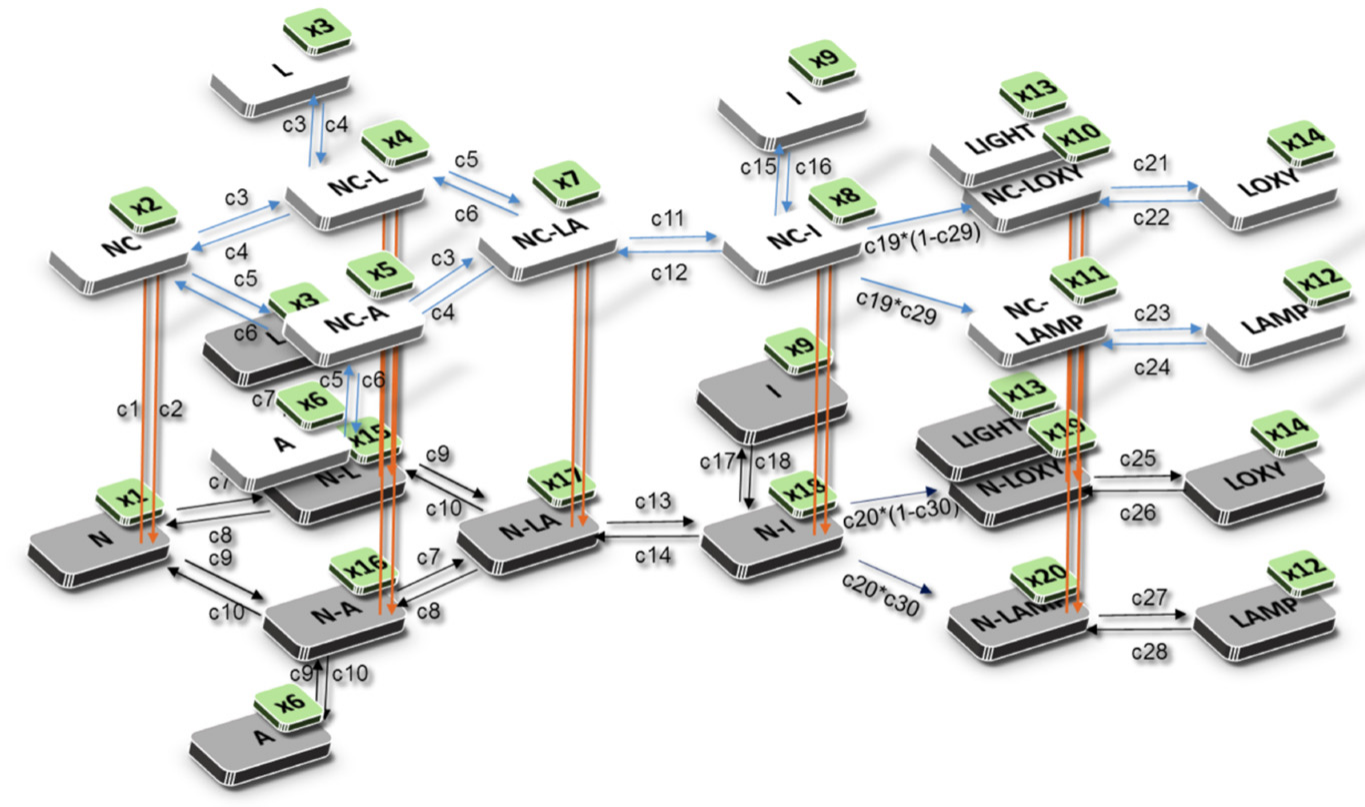
Diagram describing the complete set of reactions used to develop a mathematical model for the *in vitro* FLCA. The interaction of the protein pair (orange arrows) fused to NFLuc (grey panels) and CFLuc (not shown) forms the NC complex (white panels), which reconstitutes enzymatic activity. The reconstituted activity produces luminescence by the adenylation and oxidation of LH_2_. NFLuc contains all known substrate binding residues and can catalyze the reactions on its own [12, 13], and some luminescence can be produced without the interaction of the protein pair. The mathematical model takes into account enzymatic reaction of NFLuc alone and NC complex. The equations describing the reactions of NFLuc mirror that of the NC complex. “x” refers to variable number in the model for each species, and “c” refers to the reaction rate parameters. N: NFLuc. NC: NC complex. A: ATP. L: LH_2_. NC-A: NC bound to ATP. NC-L: NC bound to LH_2_. NC-LA: NC bound to LH_2_ and ATP. NC-I: NC bound to LH_2_-AMP. I: Free LH_2_-AMP. NC-LOXY: NC bound to L-oxy. NC-LAMP: NC bound to L-AMP. LOXY: Free L-oxy. LAMP: Free L-AMP. LIGHT: Observed luminescence. N-A: NFLuc bound to ATP. N-L: NFLuc bound to LH_2_. N-LA: NFLuc to LH_2_ and ATP. N-I: NFLuc bound to LH_2_-AMP. N-LOXY: NFLuc bound to L-oxy. N-LAMP: NFLuc bound to L-AMP.

The kinetics of a firefly luciferase mutant with a C domain deletion have been studied previously [12, 13]. The kinetics of the deleted mutant is significantly different from full length luciferase (Fig 3). In our analysis of the FLCA, it was apparent that the kinetics of the FLCA are different from both full length luciferase and the deletion mutant (Figs 2 and 3). It has been shown that NFLuc alone can bind and adenylate LH_2_ and oxidize the intermediate, although the activity is 10^−5^ fold of the full length [12]. Hence, we incorporated the independent function of NFLuc, CFLuc, and the NC complex in modeling the FLCA (Fig 4).

Our equations were written based on the following assumptions. NFLuc and CFLuc reconstitute the active site of firefly luciferase upon the association of the protein pair fused to NFLuc and CFLuc, respectively [3]. ATP and LH_2_, the substrates of firefly luciferase, can bind to NFLuc independently from CFLuc [11]. With both substrates bound, NFLuc catalyzes the adenylation and oxidation reactions, but at a much lower rate than when CFLuc is present [13, 67]. The reconstituted active site is disrupted by dissociation of the protein pair fused to NFLuc and CFLuc. The two products, L-oxy and L-AMP, inhibit luciferase competitively upon formation [6, 7]. L-oxy is the light emitter and the primary product, while L-AMP does not produce light [7].

### System of ordinary differential equations describing the FLCA

$$\frac{dx_{1}}{dt}\;=\;-c_{1}\;\cdot \;x_{1}\cdot \;x_{21}\;+\;c_{2}\;\cdot \;x_{2}\;-\;c_{9}\;\cdot \;x_{1}\;\cdot \;x_{6}\;+\;c_{10}\;\cdot \;x_{16}\;-\;c_{7}\;\cdot \;x_{1}\cdot \;x_{3}\;+\;c_{8}\;\cdot \;x_{15}\;-\;c_{26}\;\cdot \;x_{1}\;\cdot \;x_{14}\;+\;c_{25}\;\cdot \;x_{19}\;-\;c_{28}\;\cdot \;x_{1}\cdot \;x_{12}\;+\;c_{27}\;\cdot \;x_{20}\;-\;c_{15}\;\cdot \;x_{1}\;\cdot \;x_{9}\;+\;c_{16}\;\cdot \;x_{18}$$ (5)

$$\frac{dx_{2}}{dt}\;=\;c_{1}\;\cdot \;x_{1}\cdot \;x_{21}\;-\;c_{2}\;\cdot \;x_{2}\;-\;c_{3}\;\cdot \;x_{2}\;\cdot \;x_{3}\;+\;c_{4}\;\cdot \;x_{4}\;-\;c_{5}\;\cdot \;x_{2}\cdot \;x_{6}\;+\;c_{6}\;\cdot \;x_{5}\;-\;c_{23}\;\cdot \;x_{2}\;\cdot \;x_{12}\;+\;c_{24}\;\cdot \;x_{11}\;-\;c_{21}\;\cdot \;x_{2}\cdot \;x_{14}\;+\;c_{22}\;\cdot \;x_{10}\;-\;c_{15}\;\cdot \;x_{2}\;\cdot \;x_{9}\;+\;c_{16}\;\cdot \;x_{8}$$ (6)

$$\frac{dx_{3}}{dt}\;=\;-c_{3}\;\cdot \;x_{2}\cdot \;x_{3}\;+\;c_{4}\;\cdot \;x_{4}\;-\;c_{3}\;\cdot \;x_{5}\;\cdot \;x_{3}\;+\;c_{4}\;\cdot \;x_{7}\;-\;c_{7}\;\cdot \;x_{1}\cdot \;x_{3}\;+\;c_{8}\;\cdot \;x_{15}\;-\;c_{7}\;\cdot \;x_{16}\;\cdot \;x_{3}\;+\;c_{8}\;\cdot \;x_{17}$$ (7)

$$\frac{dx_{4}}{dt}\;=\;c_{3}\;\cdot \;x_{2}\cdot \;x_{3}\;-\;c_{4}\;\cdot \;x_{4}\;-\;c_{5}\;\cdot \;x_{4}\;\cdot \;x_{6}\;+\;c_{6}\;\cdot \;x_{7}\;+\;c_{1}\;\cdot \;x_{15}\cdot \;x_{21}\;-\;c_{2}\;\cdot \;x_{4}$$ (8)

$$\frac{dx_{5}}{dt}\;=\;-c_{3}\;\cdot \;x_{5}\cdot \;x_{3}\;+\;c_{4}\;\cdot \;x_{7}\;+\;c_{5}\;\cdot \;x_{2}\;\cdot \;x_{6}\;-\;c_{6}\;\cdot \;x_{5}\;+\;c_{1}\;\cdot \;x_{16}\cdot \;x_{21}\;-\;c_{2}\;\cdot \;x_{5}$$ (9)

$$\frac{dx_{6}}{dt}\;=\;-c_{5}\;\cdot \;x_{2}\cdot \;x_{6}\;+\;c_{6}\;\cdot \;x_{5}\;-\;c_{5}\;\cdot \;x_{4}\;\cdot \;x_{6}\;+\;c_{6}\;\cdot \;x_{7}\;-\;c_{9}\;\cdot \;x_{1}\cdot \;x_{6}\;+\;c_{10}\;\cdot \;x_{16}\;-\;c_{9}\;\cdot \;x_{15}\;\cdot \;x_{6}+\;c_{10}\;\cdot \;x_{17}$$ (10)

$$\frac{dx_{7}}{dt}\;=\;c_{3}\;\cdot \;x_{5}\cdot \;x_{3}\;-\;c_{4}\;\cdot \;x_{7}\;+\;c_{5}\;\cdot \;x_{4}\;\cdot \;x_{6}\;-\;c_{6}\;\cdot \;x_{7}\;+\;c_{1}\;\cdot \;x_{17}\cdot \;x_{21}\;-\;c_{2}\;\cdot \;x_{7}\;-\;c_{11}\;\cdot \;x_{7}\;+\;c_{12}\;\cdot \;x_{8}$$ (11)

$$\frac{dx_{8}}{dt}\;=\;c_{11}\;\cdot \;x_{7}\;-\;c_{12}\;\cdot \;x_{8}\;+\;c_{1}\;\cdot \;x_{18}\;\cdot \;x_{21}\;-\;c_{2}\;\cdot \;x_{8}\;-\;c_{19}\;\cdot \;x_{8}\;+\;c_{15}\;\cdot \;x_{2}\;\cdot \;x_{9}\;-\;c_{16}\;\cdot \;x_{8}$$ (12)

$$\frac{dx_{9}}{dt}\;=\;-c_{15}\;\cdot \;x_{2}\;\cdot \;x_{9}\;+\;c_{16}\;\cdot \;x_{8}\;-\;c_{17}\;\cdot \;x_{1}\;\cdot \;x_{9}\;+\;c_{18}\;\cdot \;x_{18}$$ (13)

$$\frac{dx_{10}}{dt}\;=\;c_{19}\;\cdot \;x_{8}\;\cdot \;\left(1\;-\;c_{29}\right)\;+\;c_{1}\;\cdot \;x_{19}\;\cdot \;x_{21}\;-\;c_{2}\;\cdot \;x_{10}\;+\;c_{21}\;\cdot \;x_{2}\;\cdot \;x_{14}\;-\;c_{22}\;\cdot \;x_{10}$$ (14)

$$\frac{dx_{11}}{dt}\;=\;c_{19}\;\cdot c_{29}\;\cdot \;x_{8}\;+\;c_{1}\;\cdot \;x_{20}\;\cdot \;x_{21}\;-\;c_{2}\;\cdot \;x_{11}\;+\;c_{23}\;\cdot \;x_{2}\;\cdot \;x_{12}\;-\;c_{24}\;\cdot \;x_{11}$$ (15)

$$\frac{dx_{12}}{dt}\;=\;-c_{23}\;\cdot \;x_{2}\;\cdot \;x_{12}\;+\;c_{24}\;\cdot \;x_{11}\;-\;c_{28}\;\cdot \;x_{1}\;\cdot \;x_{12}\;+\;c_{27}\;\cdot \;x_{20}$$ (16)

$$\frac{dx_{13}}{dt}\;=\;c_{19}\;\cdot \;x_{8}\;\cdot \;\left(1\;-\;c_{29}\right)\;-x_{13}\;+\;c_{20}\;\cdot \;x_{18}\;\cdot \;\left(1\;-\;c_{29}\right)$$ (17)

$$\frac{dx_{14}}{dt}\;=\;-c_{21}\;\cdot \;x_{2}\;\cdot x_{14}\;+\;c_{22}\;\cdot \;x_{10}\;-c_{26}\;\cdot \;x_{1}\;\cdot x_{14}\;+\;c_{25}\;\cdot \;x_{19}$$ (18)

$$\frac{dx_{15}}{dt}\;=\;c_{7}\;\cdot \;x_{1}\;\cdot x_{3}\;-\;c_{8}\;\cdot \;x_{15}\;-c_{9}\;\cdot \;x_{15}\;\cdot x_{6}\;+\;c_{10}\;\cdot \;x_{17}\;-\;c_{1}\;\cdot \;x_{15}\;\cdot x_{21}\;+\;c_{2}\;\cdot \;x_{4}$$ (19)

$$\frac{dx_{16}}{dt}\;=\;c_{9}\;\cdot \;x_{1}\;\cdot x_{6}\;-\;c_{10}\;\cdot \;x_{16}\;-c_{7}\;\cdot \;x_{16}\;\cdot x_{3}\;+\;c_{8}\;\cdot \;x_{17}\;-\;c_{1}\;\cdot \;x_{16}\;\cdot x_{21}\;+\;c_{2}\;\cdot \;x_{5}$$ (20)

$$\frac{dx_{17}}{dt}\;=\;-c_{13}\;\cdot \;x_{17}\;+\;c_{14}\;\cdot \;x_{18}\;+c_{9}\;\cdot \;x_{15}\;\cdot x_{6}\;-\;c_{10}\;\cdot \;x_{17}\;+\;c_{7}\;\cdot \;x_{16}\;\cdot x_{3}\;-\;c_{8}\;\cdot \;x_{17}-\;c_{1}\;\cdot \;x_{17}\;\cdot \;x_{21}\;+\;c_{2}\;\cdot \;x_{7}$$ (21)

$$\frac{dx_{18}}{dt}\;=\;c_{13}\;\cdot \;x_{17}\;-\;c_{14}\;\cdot \;x_{18}\;-c_{1}\;\cdot \;x_{18}\;\cdot x_{21}\;+\;c_{2}\;\cdot \;x_{8}\;-\;c_{20}\;\cdot \;x_{18}\;+\;c_{17}\;\cdot \;x_{1}\cdot \;x_{9}\;-\;c_{18}\;\cdot \;x_{18}$$ (22)

$$\frac{dx_{19}}{dt}\;=\;c_{20}\;\cdot \;\left(1\;-\;c_{29}\right)\;\cdot \;x_{18}\;-c_{1}\;\cdot \;x_{19}\;\cdot x_{21}\;+\;c_{2}\;\cdot \;x_{10}\;+\;c_{26}\;\cdot \;x_{1}\;\cdot \;x_{14}\;-\;c_{25}\;\cdot \;x_{19}$$ (23)

$$\frac{dx_{20}}{dt}\;=\;c_{20}\;\cdot \;c_{29}\;\cdot x_{18}\;-c_{1}\;\cdot \;x_{20}\;\cdot x_{21}\;+\;c_{2}\;\cdot \;x_{11}\;+\;c_{28}\;\cdot \;x_{1}\;\cdot \;x_{12}\;-\;c_{27}\;\cdot \;x_{20}$$ (24)

$$\frac{dx_{21}}{dt}\;=\;-c_{1}\;\cdot \;x_{21}\cdot \;x_{1}\;-\;c_{1}\;\cdot \;x_{21}\;\cdot \;x_{15}-\;c_{1}\;\cdot \;x_{21}\;\cdot \;x_{16}\;-\;c_{1}\;\cdot \;x_{21}\;\cdot \;x_{17}-\;c_{1}\;\cdot \;x_{21}\cdot \;x_{20}\;-\;c_{1}\;\cdot \;x_{21}\;\cdot \;x_{19}\;+\;c_{2}\;\cdot \;x_{2}\;+\;c_{2}\;\cdot \;x_{4}\;+\;c_{2}\;\cdot \;x_{5}\;+\;c_{2}\;\cdot \;x_{11}\;+\;c_{2}\;\cdot \;x_{10}\;+\;c_{2}\;\cdot \;x_{7}\;-\;c_{1}\;\cdot \;x_{21}\;\cdot \;x_{18}\;+\;c_{2}\;\cdot \;x_{8}$$ (25)

### Parameters were optimized by curve fitting to experimental data

We first obtained initial estimates for the parameters from the literature (Table 1). Then we attempted to estimate the adenylation rate of LH_2_, which is unknown for NFLuc. To this end, we selected ODEs that represent the binding and catalysis events of NFLuc without CFLuc in the FLCA, NFLuc alone (S2 ODE). We then applied known parameters, which were experimentally obtained in two independent studies (Table 1). The first study was conducted *in vitro* with a double mutant in the C domain of firefly luciferase [10]. The second study was conducted *in vitro* with firefly luciferase that has a nonsense mutation in the end of the N domain, resulting the loss of the C domain [12]. To estimate the adenylation rate of LH_2_, we curve fit the numerical solutions of the selected ODEs with known parameters to luminescence data that were obtained *in vitro* with the nonsense mutation of firefly luciferase (Fig 3) [13].

**Table 1:** Parameters Derived from the Literature.

**Parameters for full length luciferase**		
LH_2_ Affinity	7.2–15 *μ*M	[9, 12]
ATP Affinity	160–230 *μ*M	[9, 12]
LH_2_-AMP Affinity	4.7 *μ*M	[10]
Adenylation Rate	1⋅10^−3^ *s*^−1^	Estimated‡ [10]
Catalytic Activity	0.23 *s*^−1^	[10]
Parameters for NFLuc alone*		
LH_2_ Affinity	26–67 *μ*M	[9, 12]
ATP Affinity	560–6900 *μ*M	[9, 12]
LH_2_-AMP Affinity	0.55 *μ*M	[10]
Adenylation Rate	1⋅10^−5^ *s*^−1^	Estimated‡ [10]
Catalytic Activity	3.11⋅10^−5^ *s*^−1^	[10]
**Parameters shared between the NC complex and NFLuc alone**		
L-AMP Affinity	3.8 nM	[6, 7, 68]
L-Oxy Affinity	500 nM	[6, 7]
Dark Reaction Frequency	> 0.2	[7, 9]
Degradation of NFLuc and CFLuc	1.36⋅10^−3^ *s*^−1^	Calculated† [4]
**Parameters describing the protein pair**		
p53 and mdm2 Affinity	212 nM	[64]
*k*_*on*_ of p53 and mdm2	9.2⋅10^−3^ *nM*^−1^ *s*^−1^	[64]
*k*_*off*_ of p53:mdm2	2 *s*^−1^	[64]
Nutlin-3 and mdm2 Affinity	216–250 nM	Calculated†
FRB and Rapamycin Affinity	26 *μ*M	[69]
FKBP and Rapamycin Affinity	200 pM	[69]
FRB and FKBP:Rapamycin Affinity	12 nM	[69]

Initial estimates for parameter values were taken from the literature where available. Some values were calculated from experimental results (†) or estimated (‡) from relative rate comparisons. Values for mutant luciferases with a C domain deletion [12] or without the catalytic residues on the C domain [10] are reported here (*).

**Fig 5:**
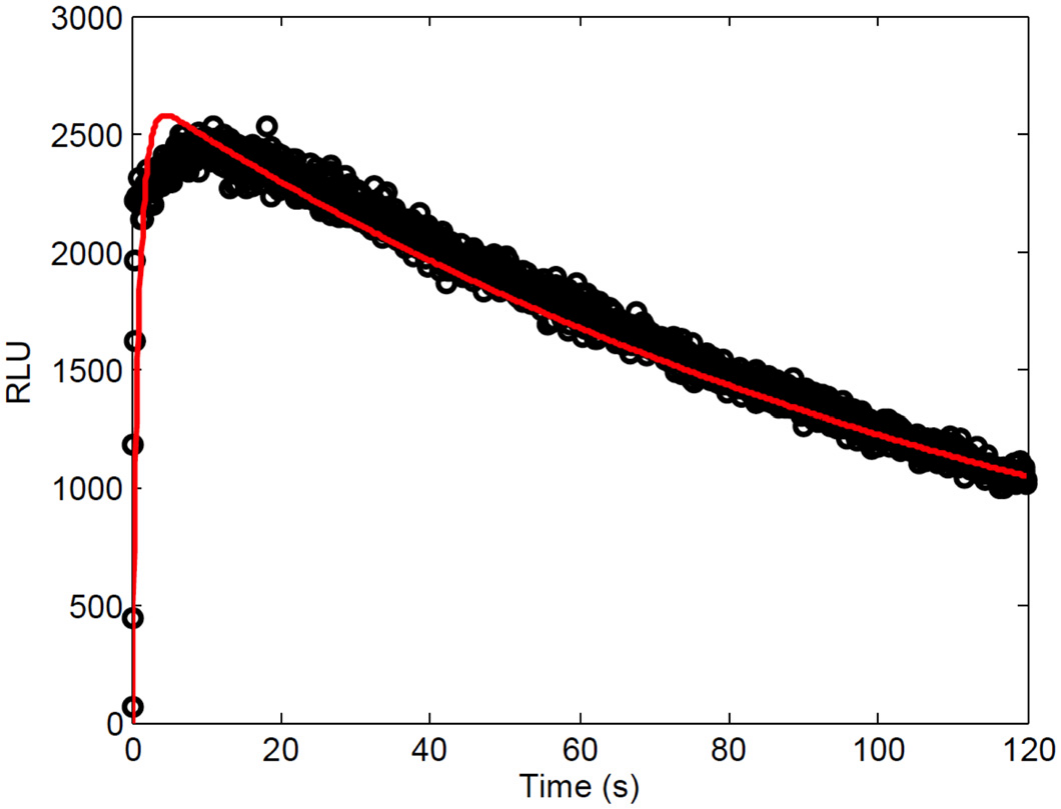
Model simulation (red) of the luminescence kinetics of p53-NFLuc and mdm2-CFLuc using *in vitro* FLCA compared to the data (black) after optimization. The values for the parameters in the mathematical model were estimated in three steps. First, parameter values were taken from the previously published literature when able, or calculated from previous data (Table 1). Second, additional parameter values were estimated by curve fitting the model to the luminescence kinetics of NFLuc alone [12] (Fig 3). Finally, the binding and catalysis rates of the NC complex were optimized by curve fitting (red) to the luminescence kinetics of 50 nM each of p53-NFLuc and mdm2-CFLuc obtained in this study (black).

The parameters were optimized using the system of ODEs representing all the enzymatic reactions occurring in the FLCA. We curve fit the numerical solutions of the ODEs to *in vitro* FLCA luminescence data (Fig 5). These data were obtained by conducting FLCA with NFLuc fused to the C terminal end of p53, and CFLuc fused to the C terminal end of mdm2 [4]. We used the dissociation constant of 212 nM for the p53-NFLuc and mdm2-CFLuc interaction that was previously obtained for the p53 and mdm2 interaction [64]. The optimized parameters of the model obtained through the curve fits are summarized in Table 2 and detailed in S1 Table.

**Table 2:** Comparison of Experimental and Estimated Values.

Parameter	Full length (experimental)†	NC complex (estimated)‡	NFLuc alone (estimated)‡	N domain (experimental)†
LH_2_ Affinity	7.2–15 *μ*M [9, 12]	16 *μ*M	27.50 *μ*M	26–67 *μ*M [9, 12]
ATP Affinity	160–230 *μ*M [9, 12]	160 *μ*M	683 *μ*M	560–6900 *μ*M [9, 12]
LH_2_-AMP Affinity	4.70 *μ*M [10]	45 nM	45 nM	550 nM [10]
L-oxy Affinity	500 nM [6, 7, 68]	70 nM	70 nM	–
L-AMP Affinity	3.80 nM [6, 7]	45 pM	45 pM	–
Adenylation Rate	–	500 *s*^−1^	0.004 *s*^−1^	–
Oxidation Rate	0.23 *s*^−1^[10]	0.22 *s*^−1^	4.00⋅10^−7^ *s*^−1^	3.11⋅10^−5^ *s*^−1^[10]
Dark Reaction Frequency	0.2 [7, 10]	0.29	0.29	> 0.2 [10]

^†^: Values obtained from the literature.

^‡^: Estimated values were obtained from the curve fit.

–: data unavailable. Values for mutant luciferases without the catalytic residues on the C domain [10] or with a C domain deletion [12] are also reported here.

### Reliability of the model was validated by simulating *in vitro* FLCA

To validate the model and optimized parameters (S1 Table), we simulated an IC-50 curve by modifying the mathematical model to reflect the three way interaction. The IC-50 can be defined as the point at which the observed binding of two proteins is decreased by 50% by an inhibitor [70]. The K_i_ is an objective measurement of the affinity of a protein and the inhibitor [65]. The IC-50 and the K_i_ are not perfectly correlated due to several factors, including competition from the protein’s partner [65]. Using Eq 2, a correction factor which takes into account the strength of binding between the target protein and its binding partner and the concentration of the proteins is therefore applied to the observed IC-50 [65].

**Fig 6:**
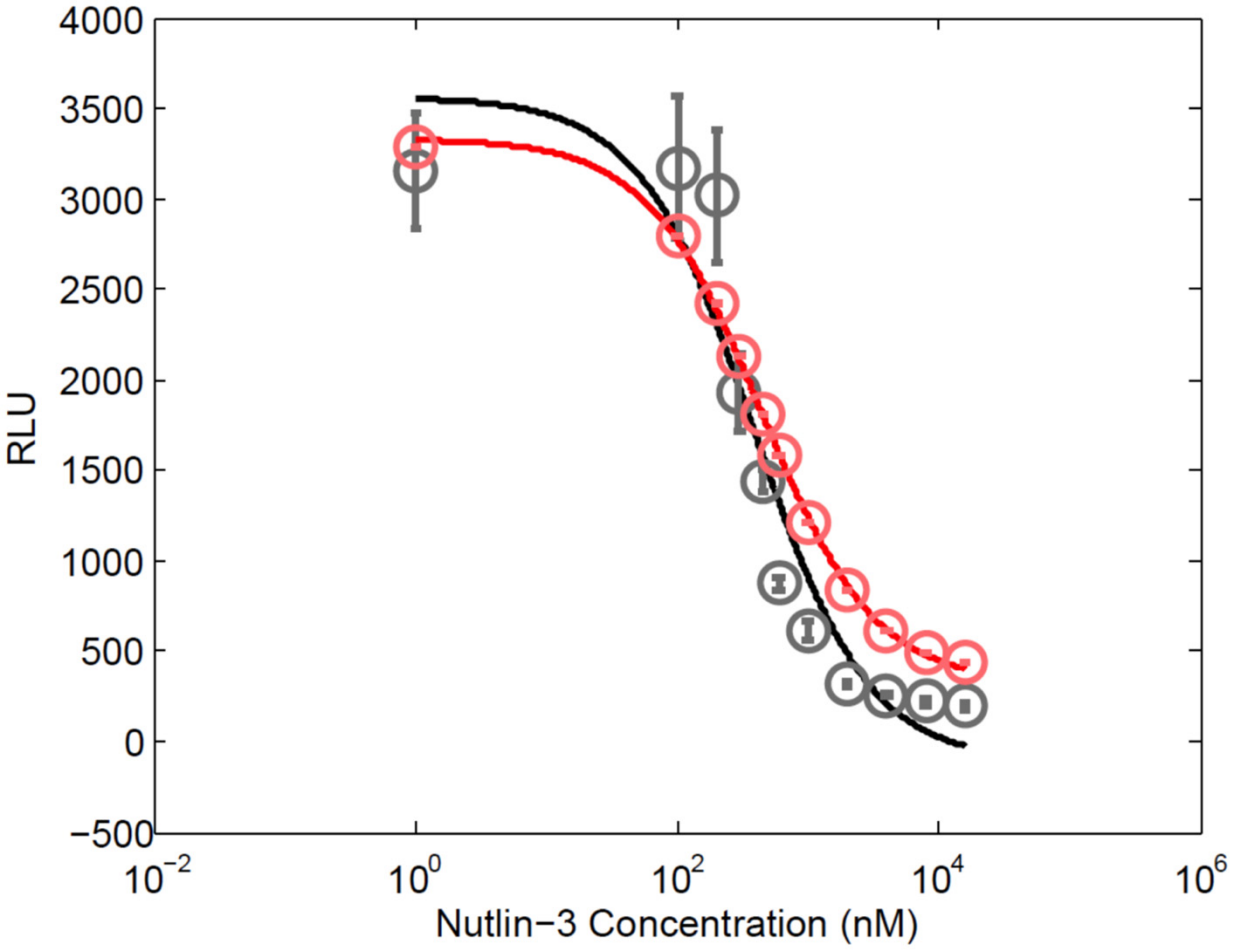
Nutlin-3 IC-50 curve (red), simulated using the *in vitro* FLCA model, agrees with the experimental data (black). Experimental RLU values of the FLCA with 100 nM each of p53-NFLuc and mdm2-CFLuc at 0.2 s (black) are compared with simulated RLU values (red) across a range of nutlin-3 concentrations [4]. The calculated IC-50 of the experimental data is 390 nM, while the simulation IC-50 is 440 nM.

Ohmuro et al. previously examined the IC-50 of nutlin-3, a specific binding inhibitor of mdm2, using the *in vitro* FLCA [4]. In the experiment, they measured the RLU immediately (0.2 sec) after adding the substrates to 100 nM each of p53-NFLuc and mdm2-CFLuc. Nutlin-3 inhibits the p53-mdm2 interaction by binding to mdm2. We estimated the K_i_ of nutlin-3 to be 235 nM using previously obtained data and Eq 2 [4]. We added ODEs describing the interaction of an inhibitor to a mdm2 fused to CFLuc (S3 ODE) to Eqs 5 through 25. Using nonlinear regression to Eq 3, we calculated the IC-50 of our simulation to be 440 nM, and the IC-50 of the previous experiment to be 390 nM (Fig 6) [70]. We assume one of the causes for the difference between the experimental data and model simulation is due to disturbances in the mixing of the substrate and enzyme solutions after injection, which would occur within the first second of the reaction. Nonetheless, the results suggest that the model and parameters of the FLCA identified in this study reasonably simulate the IC-50.

**Fig 7:**
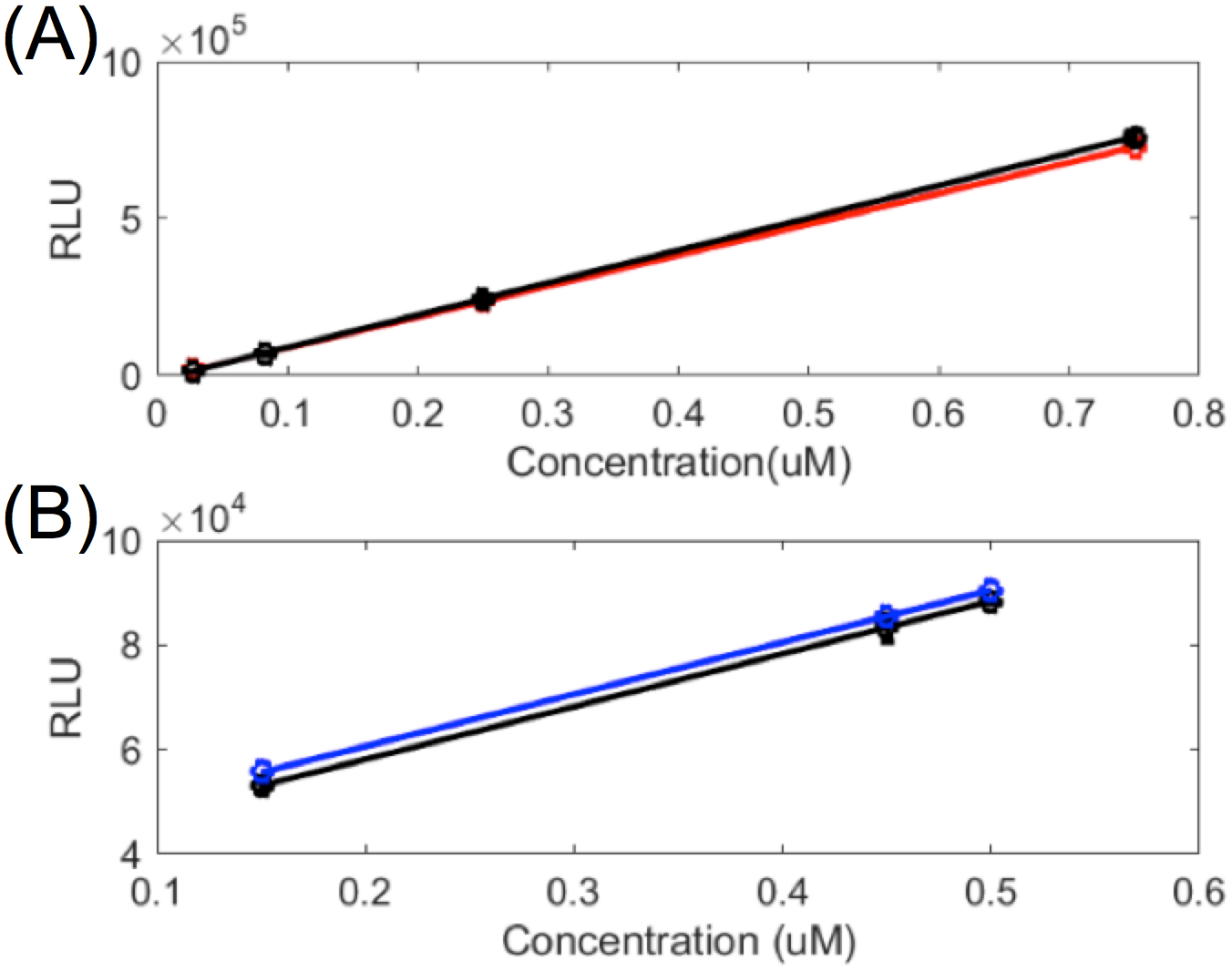
The model predicts a linear relationship between maximum RLU and concentration of the protein pair. Maximum RLU values obtained experimentally (black) are compared with simulated RLU values for (A) NFLuc-FRB, CFLuc-FKBP, and equimolar rapamycin (red) and (B) NFLuc-p53 and CFLuc-mdm2 (blue). The linear trendlines were calculated using nonlinear regression. The experimental data was obtained from [4].

As an additional validation step, we simulated the FLCA at different concentrations of NFLuc-p53 and CFLuc-mdm2 (S4 Fig). These results also suggest that the model and parameters accurately reflect the FLCA conditions, with the exception of early time points (within the first second). We assume this discrepancy is also due to the disturbance of the solution after injection of the substrate solution in the experiment. As a further validation step, we simulated the *in vitro* FLCA for a second protein pair, NFLuc-FRB and CFLuc-FKBP in the presence of equimolar rapamycin (S5 Fig). Rapamycin preferentially binds FKBP, allowing it to interact with FRB [69]. This three-way molecular interaction has a 20 times higher affinity than p53-mdm2 (Table 1). Simulating the interaction between FKBP and FRB in the presence of rapamycin using the *in vitro* FLCA with the optimized parameters reasonably agrees with the previously obtained *in vitro* FLCA data [4]. This result again suggests that the model and parameters accurately model the *in vitro* FLCA. For both NFLuc-p53:CFLuc-mdm2 and NFLuc-FRB:CFLuc-FKBP we found a linear relationship between the maximum RLU detected and the concentration of the protein pair used in the experiments (Fig 7). The model predicts that the relationship between the maximum RLU and the concentration of the protein pair will be linear (Fig 7) but the concentration affects the kinetics of light emission. Namely, the concentration of a protein pair affects the concentration of the inhibitory products, which affect the rate of light emission after the maximum RLU is reached (S6 Fig).

### The model and parameters obtained by the curve fit brought us a quantitative understanding of the luminescence production in *in vitro* FLCA

9 The model and parameters obtained in this study expanded our understanding of luminesence production in the FLCA with two important findings. First, previous experimental data showed that a mutant of the C domain residues K443 caused some changes in enzymatic activity. The mutation reduced the LH_2_-AMP oxidation rate by about 7000 times, from 0.23 *s*^−1^ in full length luciferase to 3.11⋅10^−5^ *s*^−1^ in the mutant (Table 2) [10]. On the other hand, the binding affinity for LH_2_-AMP increased by 8 times in the mutant, from 4.7 *μ*M in full length luciferase to 550 nM in the mutant (Table 2) [10]. These data indicate that K443 in the C-domain of firefly luciferase increased the oxidation rate of LH_2_-AMP while reducing the binding affinity of the N-domain for LH_2_-AMP [10].

The optimized parameters in the FLCA show a similar trend. The oxidation rate of LH_2_-AMP by NFLuc alone is 4.00⋅10^−7^ *s*^−1^ (Table 2). This is about 78 times slower than that of the mutant. On the other hand, the optimized parameters of the binding of LH_2_-AMP by NFLuc alone is 45 nM (Table 2). This is a 12 times higher affinity than that of the mutant. These parameters suggest that removing the C domain from the firefly luciferase peptide exaggerates the effects of the mutant.

Second, when the active site is reconstituted within the NC complex through the interaction of the proteins fused to NFLuc and CFLuc, the affinity of LH_2_ increases from 27.5 *μ*M in NFLuc alone to 16 *μ*M in the NC complex. This is comparable to the full length affinity to LH_2_, which has been measured between 7.2–15 *μ*M [9, 12]. The affinity of ATP also increases from 683 *μ*M in NFLuc alone to 160 *μ*M in the NC complex. This is also within the range of previously obtained full length affinity to ATP (160–230 *μ*M) [9, 12]. The oxidation rate of the NC complex was estimated to 0.22 *s*^−1^, which is very close to experimental values for full length luciferase (0.23 *s*^−1^) [10]. On the other hand, the adenylation rate of LH_2_ by the NC complex was estimated to be 500 *s*^−1^, which is about 10^5^ times higher than the value we obtained for NFLuc alone (0.004 *s*^−1^). Overall, the set of optimized parameters suggest that the NC complex reconstitutes the oxidation activity of full length firefly luciferase, while the affinities of the NC complex to LH_2_-AMP, L-oxy, and L-AMP are higher than the affinities experimentally obtained for full length firefly luciferase (Table 2).

### The mathematical model explains that the alternation of the kinetics in FLCA is due to rapid dissociation of the protein pair, lower adenylation rate, and higher affinity to LH_2_-AMP

**Fig 8:**
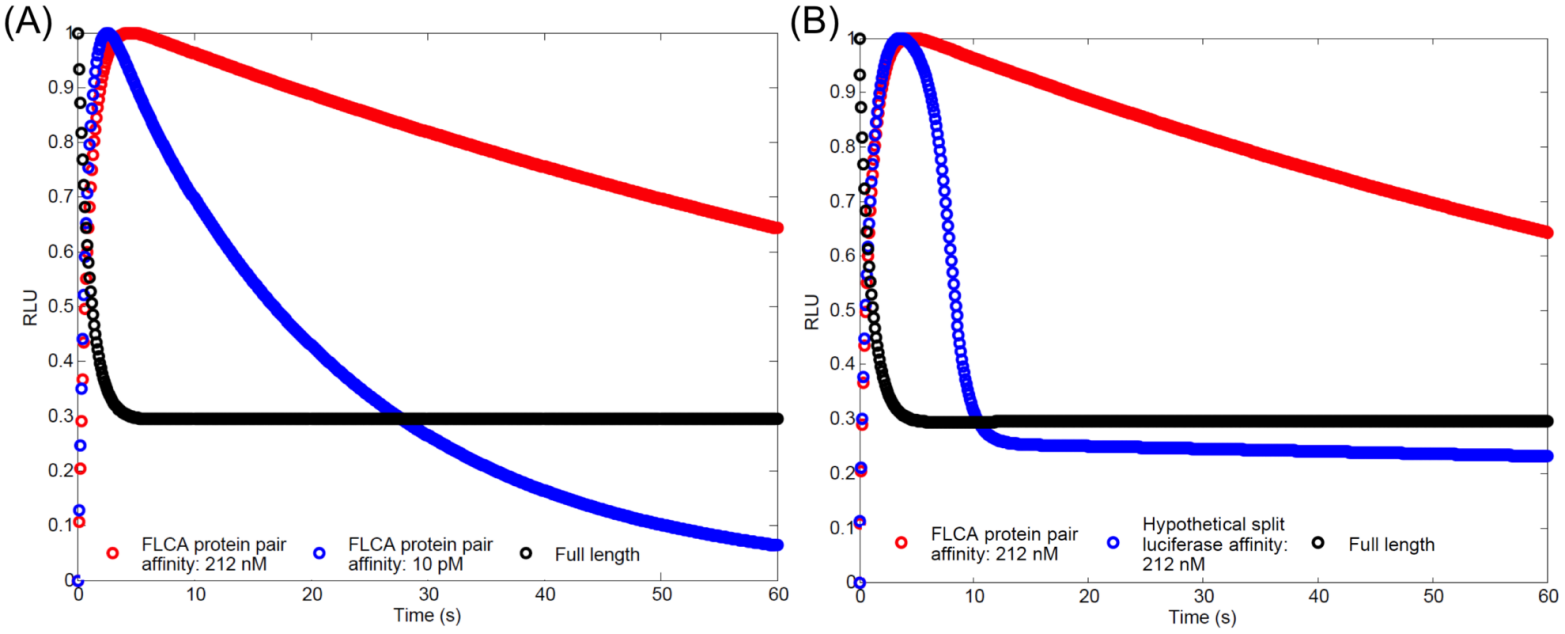
The model suggests that the delayed peak of the FLCA is due to dissociation of the protein pair, while the slow decay is due to the lower adenylation rate and higher affinity toward LH_2_-AMP. (A) Luminesence kinetics of FLCAs and full length luciferase was predicted by the model. The protein pair with K_d_ = 10 pM (blue) shows a faster peak and decay, compared with the protein pair with K_d_ = 212 nM (red). (B) Luminesence kinetics of FLCA, hypothetical split luciferase, and full length was predicted by the model. FLCA with the protein pair K_d_ of 212 nM (red) compared with a hypothetical split luciferase (blue) with the same K_d_. The hypothetical split luciferase has the same adenylation rate and affinity to LH_2_-AMP as full length luciferase. Enzyme concentration used for these simulations was 50 nM.

Although NFLuc alone has residual enzymatic activity, the oxidation activity that generates light is about 10^−6^ of the NC complex (Table 2). This suggests that the light detected in the FLCA is mainly due to the activity of the NC complex. The model estimates the oxidative rate of LH_2_-AMP in the NC complex is 0.22 *s*^−1^ while the dissociation of p53 and mdm2, fused to NFLuc and CFLuc respectively, is 2.0 *s*^−1^[64]. This suggests the active site formed in the NC complex dissociates about 10 times faster than the rate of LH_2_-AMP oxidization. The model suggests that this causes the delayed peak in the FLCA (Fig 8(A)). For example, a hypothetical protein pair with a K_d_ of 10 pM dissociates at a rate of approximately 1⋅10^−4^ *s*^−1^[63]. The model suggests that such a protein pair reaches its peak RLU around at 2.5 sec, while a protein pair with K_d_ of 212 nM (p53-mdm2) reaches the peak around at 5.0 sec.

The model further suggests that two factors cause the slow decay of the FLCA: the lower adenylation rate (estimated to be 500 *s*^−1^ for the NC complex, and 5000 *s*^−1^ for full length luciferase), and a higher affinity to LH_2_-AMP. To demonstrate this, we simulated the kinetics of full length firefly luciferase, the FLCA, and a hypothetical split luciferase that has full length values for the adenylation rate and the affinity and LH_2_-AMP, and FLCA values for all other parameters (Fig 8(B)). The hypothetical split luciferase shows similar kinetics to the full length, although the peak is still delayed.

### The mathematical model reveals maximum RLU detected in the FLCA would underestimate changes in K_d_ of an interacting protein pair

Although the observed RLU in the FLCA is expected to correlate with the degree of interaction of the protein pair, it is unknown how direct this correlation is. To address the question, the relationship between changes in affinity of a protein pair and RLU was analyzed using the mathematical model and parameters obtained in this study.

**Fig 9:**
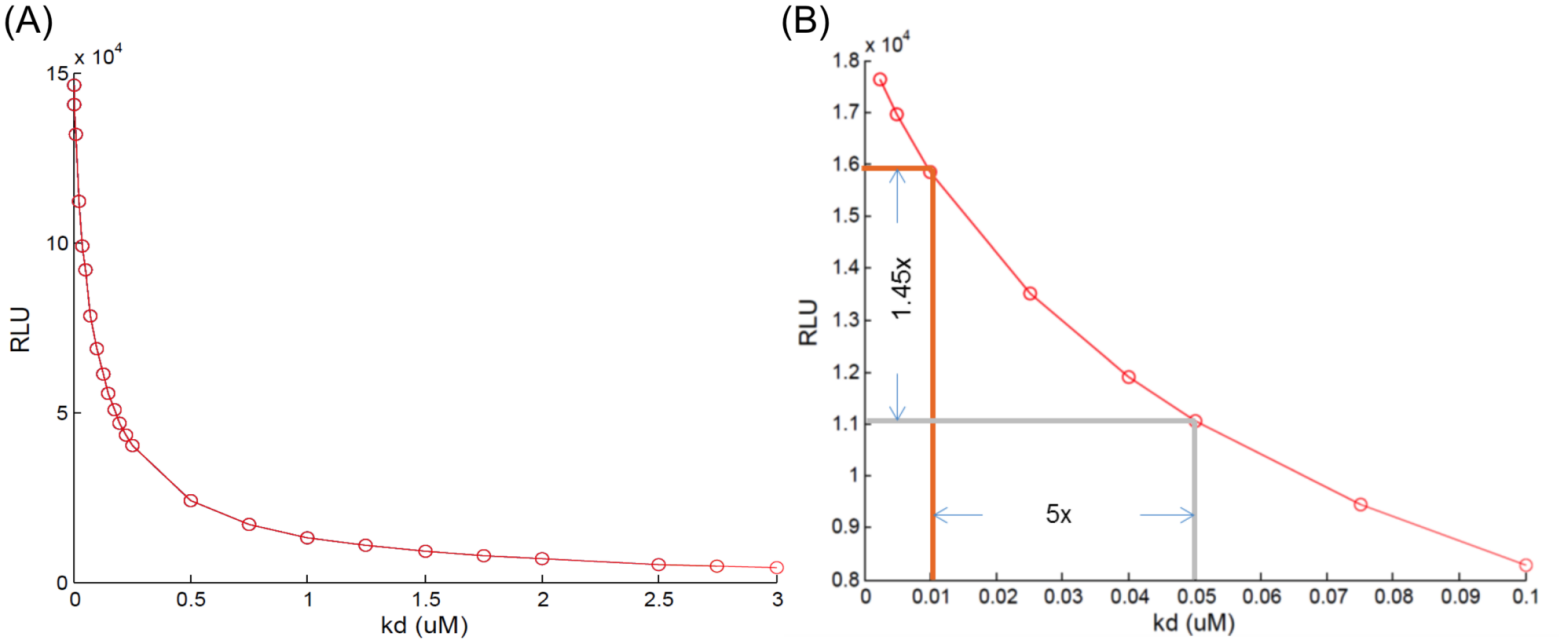
Directly comparing the RLU obtained by the FLCA may cause misunderstandings about their affinities. (A) Predicted relationship between changes of K_d_ (from 2.5 nM to 3 *μ*M) for a protein pair and the RLUs detected in the FLCA. The model predicts an exponential relationship between changes of K_d_ and maximum RLU. (B) Predicted relationship between changes of K_d_ (from 10 nM to 50 nM) for a protein pair and the RLUs detected in the FLCA. Notice the RLU detected at 10 nM (orange grid) is only 1.45 times higher that that at 50 nM (grey grid). Enzyme concentration used for these simulations was 50 nM.

The model suggests that the relationship between the K_d_ (the affinity of a protein pair) and the RLU is exponentially rather than linearly correlated. To demonstrate the relationship, we evaluated protein pair affinities over 24 different values, from 2.5 nM to 3 *μ*M (Fig 9(A)). This simulation demonstrates that the comparison of the RLU obtained using FLCA can be misleading about changes of the affinity of the protein pair. For example, when comparing the maximum RLU at 10 nM and 50 nM affinity, there is a 5 times decrease in the protein pair affinity, but only a 1.45 times decrease in the maximum RLU (Fig 9(B)). This suggests that the FLCA would underestimate changes in the K_d_ of protein pairs.

This finding raises a question about the most valid interpretation of the FLCA data. Some previously published papers claim that the K_d_ of the protein pair has a direct, linear relationship with the RLU detected with *in cellulo* FLCA [1, 14]. It is conceivable that more variables and parameters are needed to construct a mathematical model of *in cellulo* FLCA. The variables specific for *in cellulo* include protein synthesis and degradation, diffusion rates of the substrates across cell membranes, and the conversion of L-AMP to dehydroluciferyl-Coenzyme A by Coenzyme A [71]. However, one can expect the same exponential relationship between the RLU and the K_d_ *in cellulo*. We, therefore, suggest the FLCA data be interpreted as a qualitative measure of protein pair affinity until a detailed analysis or control experiment can be conducted for the *in cellulo* FLCA.

### Conclusion

In this study, we found that the change in luminescence kinetics between the FLCA and full length luciferase is due primarily to the rapid dissociation of the protein pair fused to NFLuc and CFLuc, a lower adenylation rate, and an increased affinity of the NC complex to LH_2_-AMP. Branchini et al. first demonstrated the effect of the removal of the catalytic residues of the C terminal domain on firefly luciferase by measuring the luminescence production in a recombinant protein [10]. The model constructed in this study demonstrates that the relationship between luminescence and the affinity of the protein pair fused to NFLuc and CFLuc is exponential rather than linear. The model explains that, due to the non-linear relationship between the RLU and K_d_ of a protein pair, a single luminesence value cannot be a direct quantitative measurement of affinity or protein pair complex.

One of the most challenging aspects in the FLCA when comparing the affinities of different protein pairs is to understand how the active site in a NC complex is reconstituted. It is known that when NFLuc and CFLuc are fused to different locations of a protein pair (.i.e., amino- or carboxyl- terminal end of proteins of interest via a linker peptide), the RLU observed in the FLCA assay varies greatly [4]. This suggests that the geometry of NFLuc and CFLuc in the interacting protein pair influences how the active site is reconstituted. In other words, NFLuc and CFLuc may not be able to reconstitute the fully active NC complex, depending on their geometry. This phenomenon particularly demonstrates that direct comparison of RLUs between different protein pairs, especially when the 3D structures of the proteins are largely different, must be considered qualitative. Quantitative analysis with the FLCA is possible, as demonstrated previously by Ohmuro et al. with their IC-50 analysis and our mathematical model in this study [4]. We suggest that the titration of known concentrations of CFLuc to a small concentration of NFLuc, such as for a traditional protein interaction assay, is required to obtain quantitative results for *in vitro* FLCA [72].

## Supporting Information

S1 Fig Kinetics of full length firefly luciferase is independent of concentration. (A) Kinetics at 150 nM of firefly luciferase. (B) Kinetics at 450 nM of firefly luciferase.
(TIF)

S2 Fig Determination of the degradation rate of NFLuc and CFLuc at 37°C. Previously the heat stability of NFLuc and CFLuc was analyzed by measuring the activity after incubation times ranging from 0 to 60 minutes at 37°C [4]. To calculate the degradation rate, the RLU values were digitized using PlotDigitizer and the maximum RLU values were extracted [61]. The RLU value for no incubation time was considered 100% activity. This was curve fit to an equation describing degradation (Eq 1). The degradation rate was found to be 0.00136 *s*^−1^.
(TIF)

S3 Fig Determination of the initial concentration of NC complex. Initial concentration of free NFLuc-p53, free CFLuc-mdm2, and NC complex was modeled using the affinity for p53 and mdm2 from the literature [64]. (A) For luminescence kinetic data with incubation at 37°C (shown in Figs 2 and 5), the degradation rate was included in the calculation of initial conditions. (B) For luminescence kinetics data without any incubation (see S4 Fig), probes were not incubated, but an average experimental delay of approximately 1 s is assumed.
(TIF)

S4 Fig The mathematical model (red) reasonably matches experimental data (black) at varying concentrations of NFLuc-p53 and CFLuc-mdm2. (A) 50 nM of NFLuc-p53 and CFLuc-mdm2 each. (B) 150 nM of NFLuc-p53 and CFLuc-mdm2 each. (C) 450 nM of NFLuc-p53 and CFLuc-mdm2 each. Each simulation was separately optimized with respect to the effects of the detection lens (photomultiplier tube). Data obtained from [4].
(TIF)

S5 Fig The mathematical model (red) reasonably matches experimental data (black) at varying concentrations of NFLuc-FRB and CFLuc-FKBP:rapamycin. (A) 28 nM of NFLuc-FRB and CFLuc-FKBP:rapamycin each. (B) 83 nM of NFLuc-FRB and CFLuc-FKBP:rapamycin each. (C) 250 nM of NFLuc-FRB and CFLuc-FKBP:rapamycin each. (D) 750 nM of NFLuc-FRB and CFLuc-FKBP:rapamycin each. Each simulation was separately optimized with respect to the effects of the detection lens (photomultiplier tube). Data obtained from [4].
(TIF)

S6 Fig The mathematical model predicts the concentration of the protein pair affects the kinetics. The concentration of a protein pair with a K_d_ of 100 nM was varied from 5 nM to 15 *μ*M. The concentration of the protein pair affects the amount of inhibitory products in solution after the light emission peak is reached. When the concentration is higher than the K_d_ of the protein pair, a clear peak (maximum RLU) will be most easily detectable in the kinetics. Green is the 100 nM concentration simulation.
(TIF)

S1 Table Parameter values after optimization by curve fitting mathematical model to kinetic data. Protein pair used in obtaining the experimental data for the curve fit was p53 and mdm2. Parameter estimations were obtained to 15 decimal points and rounded to the nearest hundreth.
(PDF)

S2 Table Parameter values obtained from the literature. Dissociation rates (k_off_) calculated by holding k_on_ values at those found after optimization for the FLCA model.
(PDF)

S1 ODE System of equations describing the interaction and degradation of two proteins (*x*_1_ and *x*_2_) and the interacting pair (*x*_3_). Used to calculate the initial concentrations prior to substrate addition.
(PDF)

S2 ODE System of ODEs describing the binding and catalysis of NFLuc only. These equations were obtained using knowledge of the enzymatic activity of NFLuc, as shown by the literature. For the purposes of publication, the numbering system was conserved between the full *in vitro* FLCA ODEs by removing the portions involving interaction with CFLuc.
(PDF)

S3 ODE System of equations describing the binding of an inhibitor (here, nutlin-3) to a protein attached to CFLuc (mdm2). These equations were added to the *in vitro* FLCA ODEs to model the IC-50 data obtained when adding nutlin-3 to NFLuc-p53 and CFLuc-mdm2.
(PDF)

S4 ODE Here the *in vitro* FLCA ODEs were stripped down to be able to represent full-length luciferase. Accordingly, only the interaction and catalysis of the NC complex is modeled here, with all references to free NFLuc or CFLuc removed.
(PDF)

S1 Code Matlab code used to generate long and short term simulations of NFLuc-p53 and CFLuc-mdm2, and compare it to the data. (PDF)
