# Supplementary figures and images for: Mathematical Model of the Firefly Luciferase Complementation Assay Reveals a Non-Linear Relationship between the Detected Luminescence and the Affinity of the Protein Pair Being Analyzed
Source: PLoS One. 2016 Feb 17;11(2):e0148256. doi: 10.1371/journal.pone.0148256 (PMC4757408; doi:10.1371/journal.pone.0148256)

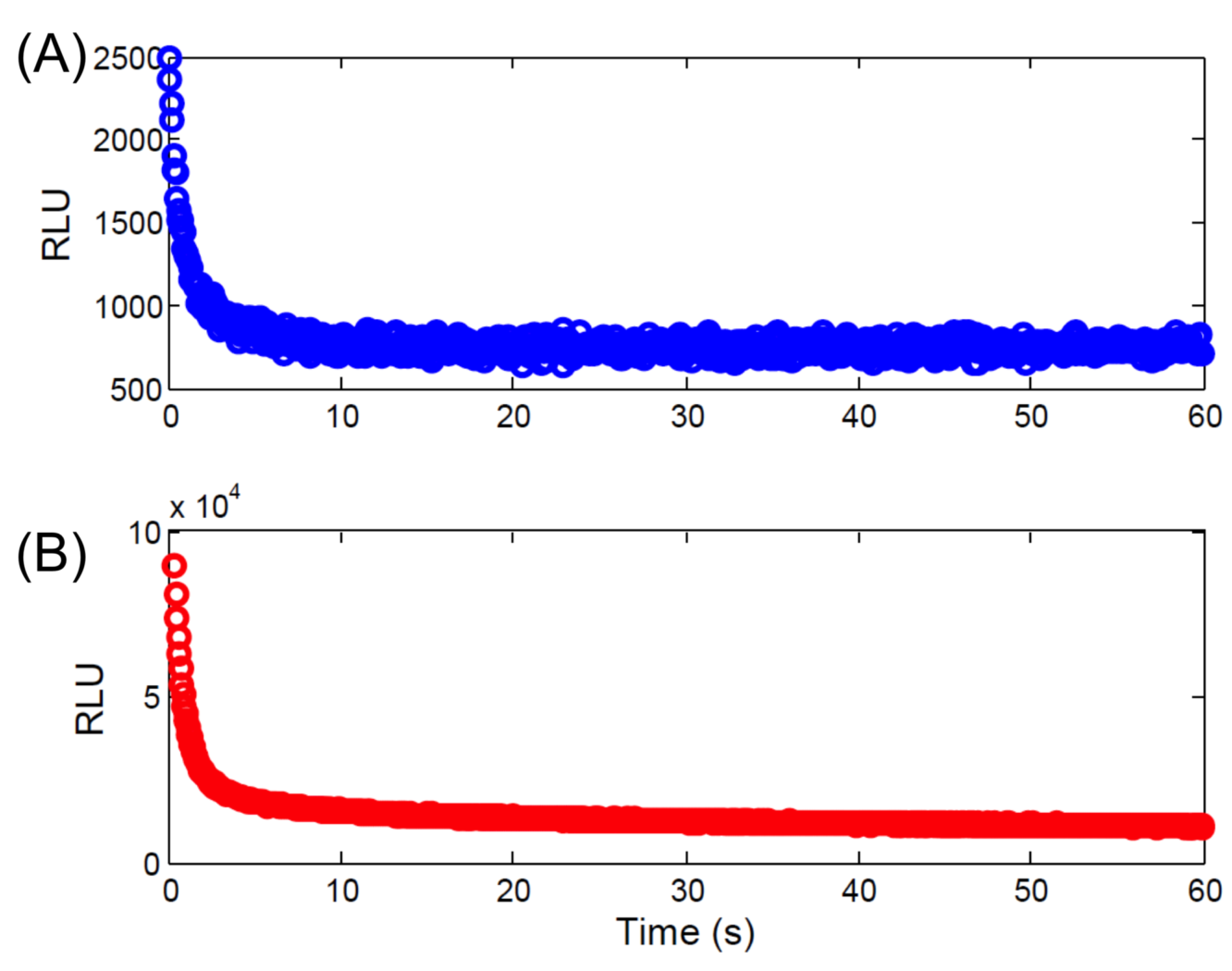

Supplement: S1 Fig — (A) Kinetics at 150 nM of firefly luciferase. (B) Kinetics at 450 nM of firefly luciferase. (TIF) [file pone.0148256.s001.tif]

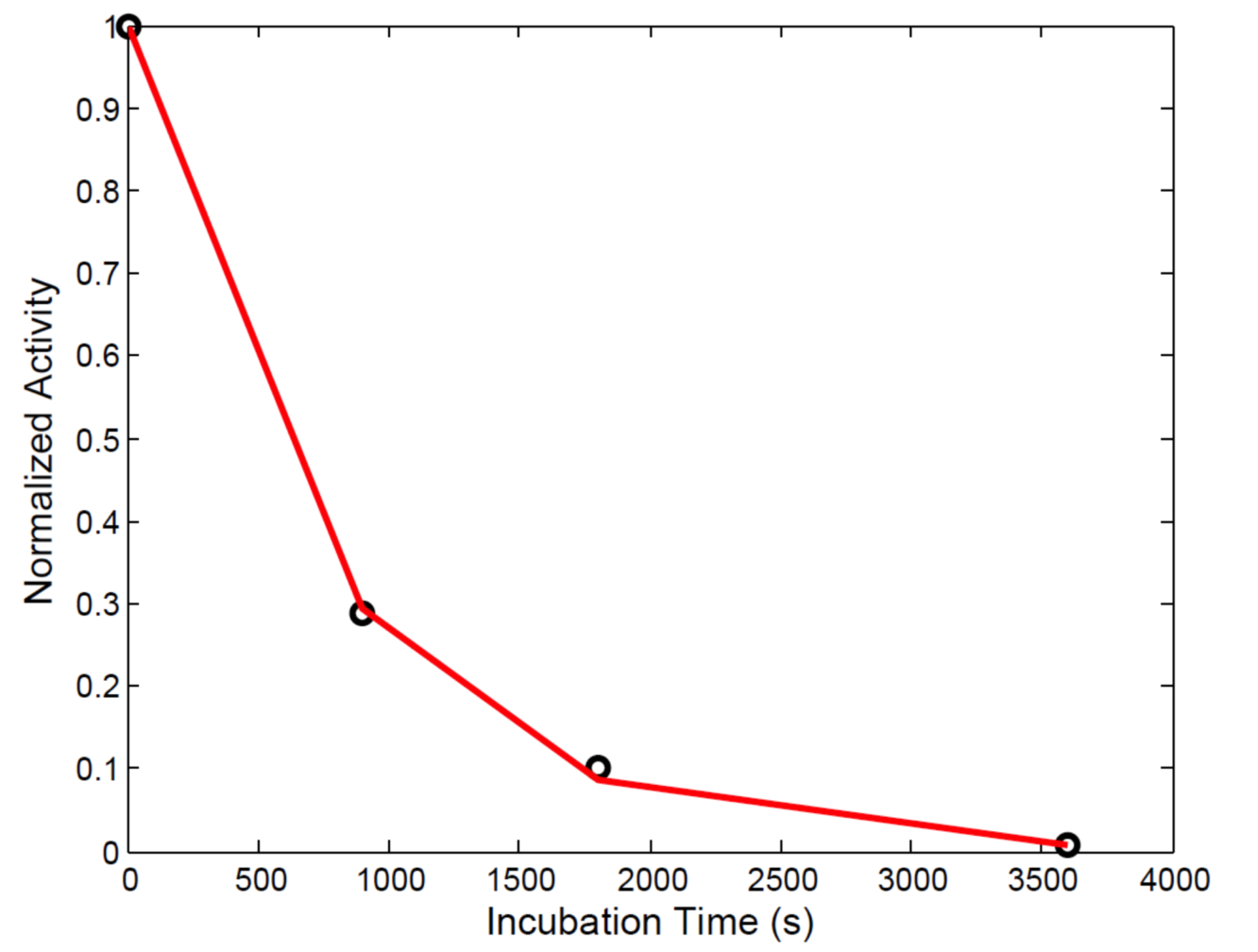

Supplement: S2 Fig — Previously the heat stability of NFLuc and CFLuc was analyzed by measuring the activity after incubation times ranging from 0 to 60 minutes at 37°C [4]. To calculate the degradation rate, the RLU values were digitized using PlotDigitizer and the maximum RLU values were extracted [61]. The RLU value for no incubation time was considered 100% activity. This was curve fit to an equation describing degradation (Eq 1). The degradation rate was found to be 0.00136 s−1. (TIF) [file pone.0148256.s002.tif]

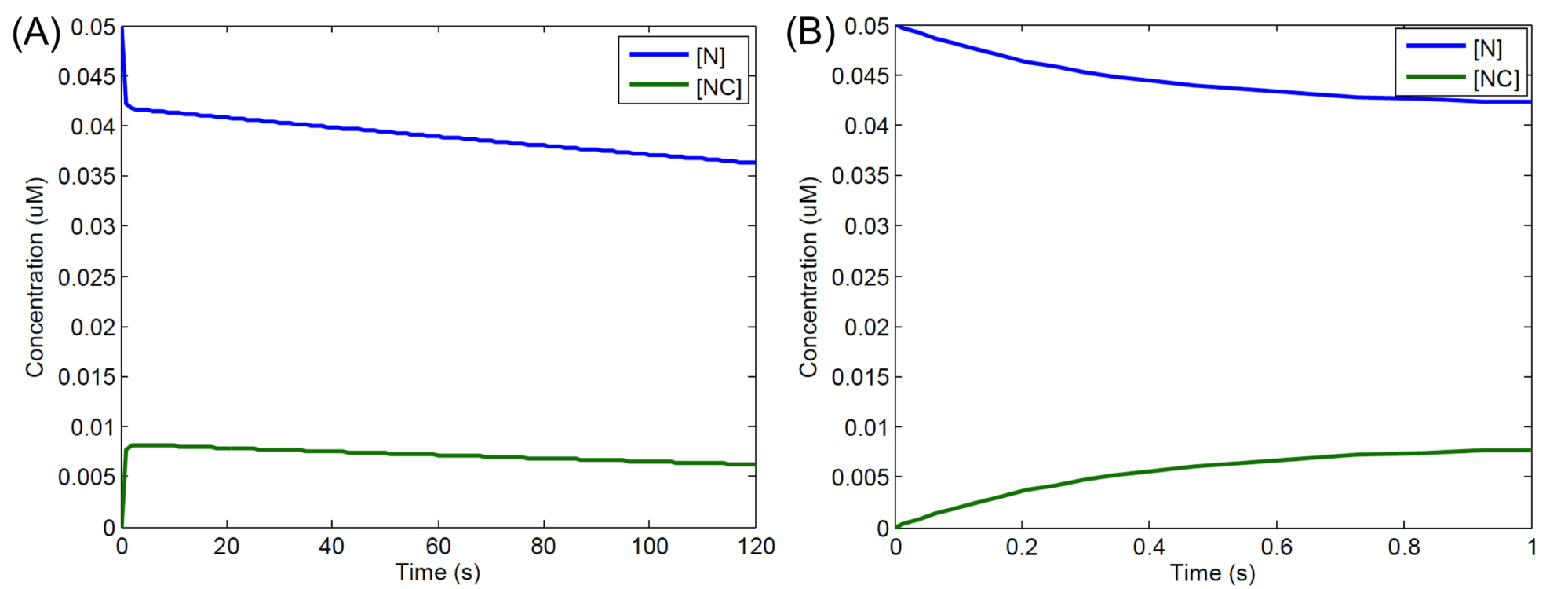

Supplement: S3 Fig — Initial concentration of free NFLuc-p53, free CFLuc-mdm2, and NC complex was modeled using the affinity for p53 and mdm2 from the literature [64]. (A) For luminescence kinetic data with incubation at 37°C (shown in Figs 2 and 5), the degradation rate was included in the calculation of initial conditions. (B) For luminescence kinetics data without any incubation (see S4 Fig), probes were not incubated, but an average experimental delay of approximately 1 s is assumed. (TIF) [file pone.0148256.s003.tif]

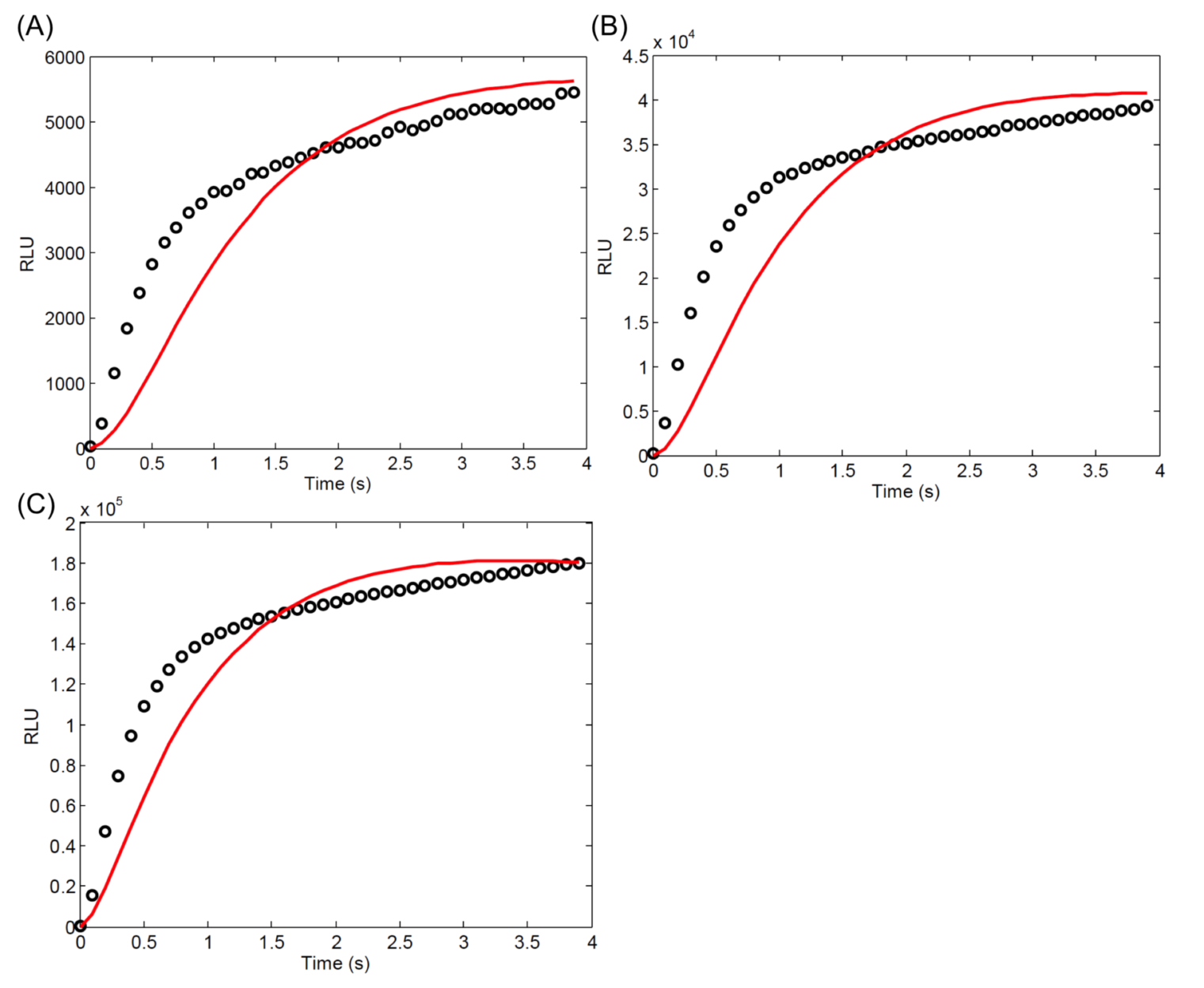

Supplement: S4 Fig — (A) 50 nM of NFLuc-p53 and CFLuc-mdm2 each. (B) 150 nM of NFLuc-p53 and CFLuc-mdm2 each. (C) 450 nM of NFLuc-p53 and CFLuc-mdm2 each. Each simulation was separately optimized with respect to the effects of the detection lens (photomultiplier tube). Data obtained from [4]. (TIF) [file pone.0148256.s004.tif]

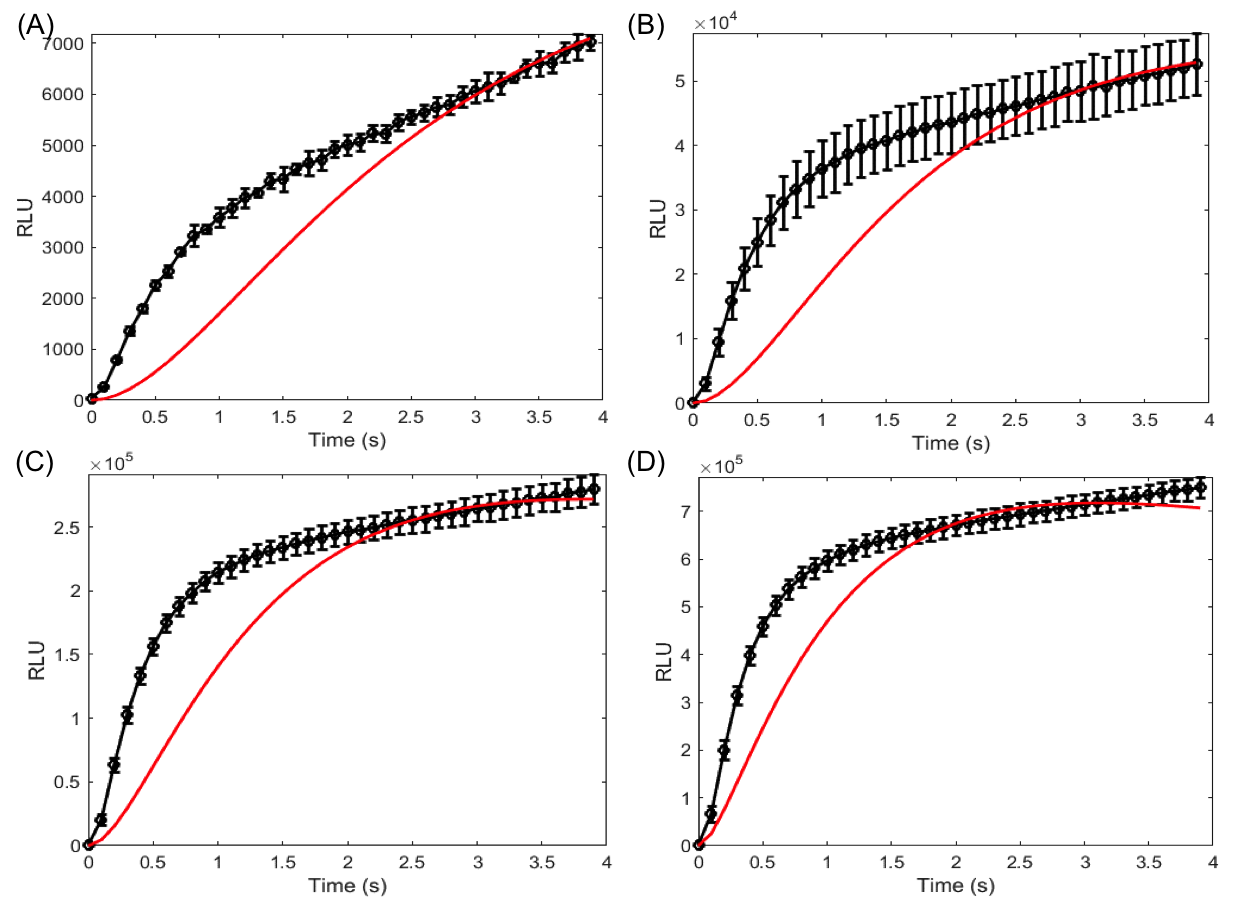

Supplement: S5 Fig — (A) 28 nM of NFLuc-FRB and CFLuc-FKBP:rapamycin each. (B) 83 nM of NFLuc-FRB and CFLuc-FKBP:rapamycin each. (C) 250 nM of NFLuc-FRB and CFLuc-FKBP:rapamycin each. (D) 750 nM of NFLuc-FRB and CFLuc-FKBP:rapamycin each. Each simulation was separately optimized with respect to the effects of the detection lens (photomultiplier tube). Data obtained from [4]. (TIF) [file pone.0148256.s005.tif]

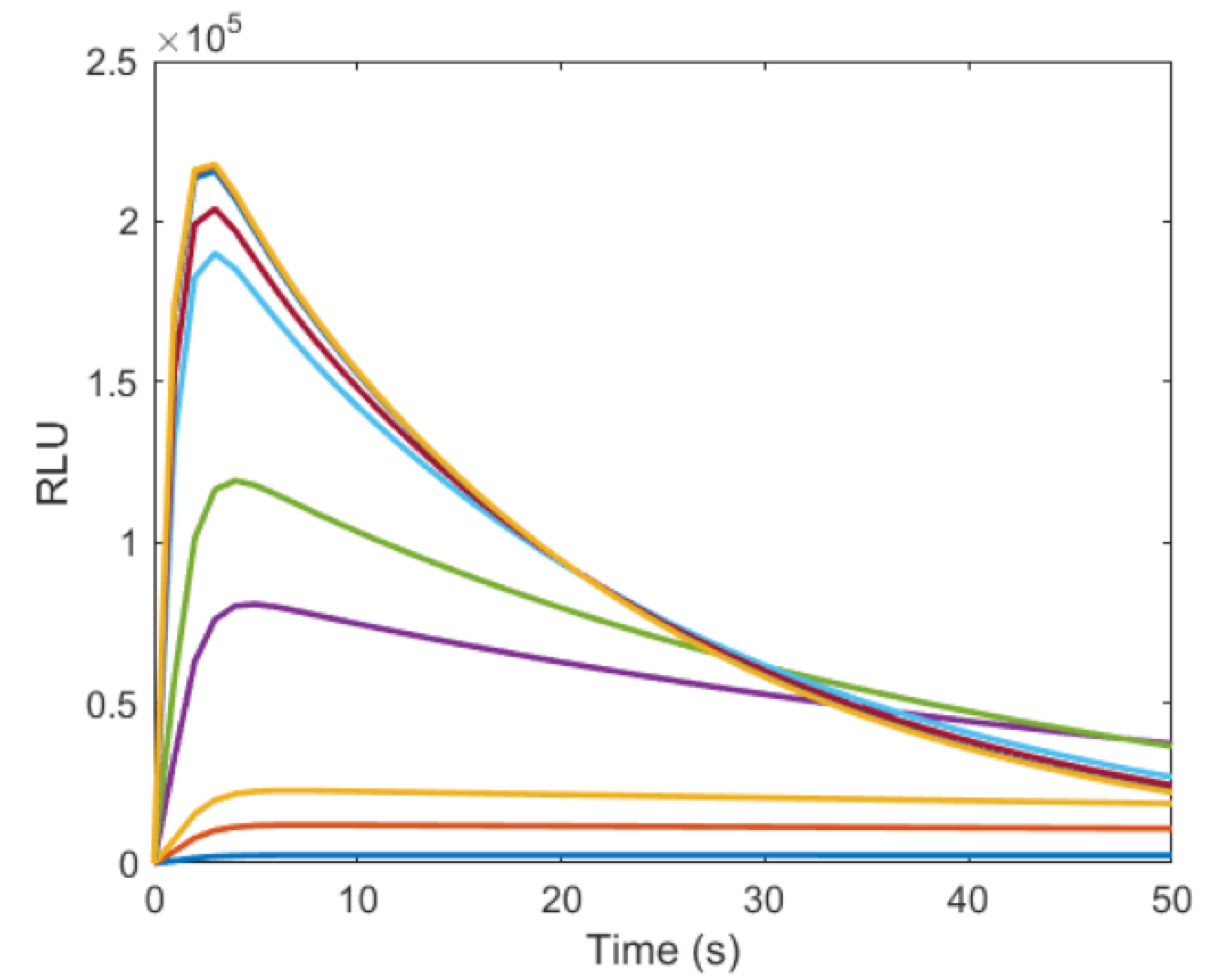

Supplement: S6 Fig — The concentration of a protein pair with a Kd of 100 nM was varied from 5 nM to 15 μM. The concentration of the protein pair affects the amount of inhibitory products in solution after the light emission peak is reached. When the concentration is higher than the Kd of the protein pair, a clear peak (maximum RLU) will be most easily detectable in the kinetics. Green is the 100 nM concentration simulation. (TIF) [file pone.0148256.s006.tif]
