# Supplementary material for: Mathematical Model of the Firefly Luciferase Complementation Assay Reveals a Non-Linear Relationship between the Detected Luminescence and the Affinity of the Protein Pair Being Analyzed
Source: PLoS One. 2016 Feb 17;11(2):e0148256. doi: 10.1371/journal.pone.0148256 (PMC4757408; doi:10.1371/journal.pone.0148256)
Supplement: S1 Table — Protein pair used in obtaining the experimental data for the curve fit was p53 and mdm2. Parameter estimations were obtained to 15 decimal points and rounded to the nearest hundreth. (PDF) [file pone.0148256.s007.pdf]

Parameters Values from the Curve Fit.

c_1	$K_{d,f}$	Association of P53 and MDM_2	$9.20{\cdot}10^6$	$M^{-1}s^{-1}$
c_2	$K_{d,r}$	Dissociation of P53 and MDM_2	2.00	s^{-1}
c_3	ka_f	Association of LH_2 to NC Complex	$1.84{\cdot}10^8$	$M^{-1}s^{-1}$
c_4	ka_r	Dissociation of LH_2 from NC Complex	$3.00{\cdot}10^3$	s^{-1}
c_5	kl_f	Association of ATP to NC Complex	$3.00{\cdot}10^7$	$M^{-1}s^{-1}$
c_6	kl_r	Dissociation of ATP from NC Complex	$4.80{\cdot}10^3$	s^{-1}
c_7	kl_{f2}	Association of LH_2 to NFLuc	$1.84{\cdot}10^8$	$M^{-1}s^{-1}$
c_8	kl_{r2}	Dissociation of LH_2 from NFLuc	$5.05{\cdot}10^3$	s^{-1}
c_9	ka_{f2}	Association of ATP to NFLuc	$3.00{\cdot}10^7$	$M^{-1}s^{-1}$
c_{10}	ka_{r2}	Dissociation of ATP from NFLuc	$2.05{\cdot}10^4$	s^{-1}
c_{11}	k_{Af}	Adenylation rate, forward, NC complex	$5.01{\cdot}10^2$	s^{-1}
c_{12}	k_{Ar}	Adenylation rate, reverse, NC complex	$1.08{\cdot}10^{-2}$	s^{-1}
c_{13}	k_{Af2}	Adenylation rate, forward, NFLuc	$5.00{\cdot}10^{-2}$	s^{-1}
c_{14}	k_{Ar2}	Adenylation rate, reverse, NFLuc	$1.10{\cdot}10^{-2}$	s^{-1}
c_{15}	kI_f	Association of Intermediate, NC complex	$7.77{\cdot}10^7$	$M^{-1}s^{-1}$
c_{16}	kI_r	Dissociation of Intermediate, NC complex	3.47	s^{-1}
c_{17}	kI_{f2}	Association of Intermediate, NFLuc	$7.77{\cdot}10^7$	$M^{-1}s^{-1}$
c_{18}	kI_{r2}	Dissociation of Intermediate, NFLuc	3.47	s^{-1}
c_{19}	k_{cat}	Oxidation Rate, NC complex	$2.1{\cdot}10^{-1}$	s^{-1}
c_{20}	k_{cat2}	Oxidation Rate, NFLuc	$4.00{\cdot}10^{-7}$	s^{-1}
c_{21}	ki_f	Association of Oxyluciferin to NC Complex	$8.30{\cdot}10^6$	$M^{-1}s^{-1}$
c_{22}	ki_r	Dissociation of Oxyluciferin from NC Complex	$6.13{\cdot}10^{-1}$	s^{-1}
c_{23}	ki_f^2	Association of L-AMP to NC Complex	$5.00{\cdot}10^7$	$M^{-1}s^{-1}$
c_{24}	ki_r^2	Dissociation of L-AMP from NC Complex	$2.30{\cdot}10^{-5}$	s^{-1}
c_{25}	ki_{f2}	Association of Oxyluciferin to NFLuc	$8.30{\cdot}10^6$	$M^{-1}s^{-1}$
c_{26}	ki_{r2}	Dissociation of Oxyluciferin from NFLuc	$6.13{\cdot}10^{-1}$	s^{-1}
c_{27}	ki_{f2}^2	Association of L-AMP to NFLuc	$5.00{\cdot}10^7$	$M^{-1}s^{-1}$
c_{28}	ki_{r2}^2	Dissociation of L-AMP from NFLuc	$2.30{\cdot}10^{-5}$	s^{-1}
c_{29}		Dark Reaction Frequency	$2.87{\cdot}10^{-1}$	
c_{30}		Heat Degradation	$1.00{\cdot}10^{-3}$	s^{-1}
