# Supplementary material for: Mathematical Model of the Firefly Luciferase Complementation Assay Reveals a Non-Linear Relationship between the Detected Luminescence and the Affinity of the Protein Pair Being Analyzed
Source: PLoS One. 2016 Feb 17;11(2):e0148256. doi: 10.1371/journal.pone.0148256 (PMC4757408; doi:10.1371/journal.pone.0148256)
Supplement: S2 Table — Dissociation rates (koff) calculated by holding kon values at those found after optimization for the FLCA model. (PDF) [file pone.0148256.s008.pdf]

Full length parameters.

c_3	ka_f	Association of LH_2	$1.84{\cdot}10^8$	$M^{-1}s^{-1}$
c_4	ka_r	Dissociation of LH_2	$1.32{\cdot}10^4$	s^{-1}
c_5	kl_f	Association of ATP	$3.00{\cdot}10^7$	$M^{-1}s^{-1}$
c_6	kl_r	Dissociation of ATP	$4.80{\cdot}10^4$	s^{-1}
c_{11}	k_{Af}	Adenylation rate, forward	$5.00{\cdot}10^3$	s^{-1}
c_{12}	k_{Ar}	Adenylation rate, reverse	$1.08{\cdot}10^{-2}$	s^{-1}
c_{15}	kI_f	Association of Intermediate	$7.77{\cdot}10^7$	$M^{-1}s^{-1}$
c_{16}	kI_r	Dissociation of Intermediate	$3.65{\cdot}10^2$	s^{-1}
c_{19}	k_{cat}	Oxidation Rate	$2.30\dot{1}0^{-1}$	s^{-1}
c_{21}	ki_f	Association of Oxyluciferin	$8.30{\cdot}10^6$	$M^{-1}s^{-1}$
c_{22}	ki_r	Dissociation of Oxyluciferin	4.15	s^{-1}
c_{23}	ki_f^2	Association of L-AMP	$5.00{\cdot}10^7$	$M^{-1}s^{-1}$
c_{24}	ki_r^2	Dissociation of L-AMP	$1.90{\cdot}10^{-1}$	s^{-1}
c_{29}		Dark Reaction Frequency	$2.00{\cdot}10^{-1}$	
