# Supplementary material for: Mathematical Model of the Firefly Luciferase Complementation Assay Reveals a Non-Linear Relationship between the Detected Luminescence and the Affinity of the Protein Pair Being Analyzed
Source: PLoS One. 2016 Feb 17;11(2):e0148256. doi: 10.1371/journal.pone.0148256 (PMC4757408; doi:10.1371/journal.pone.0148256)
Supplement: S1 ODE — Used to calculate the initial concentrations prior to substrate addition. (PDF) [file pone.0148256.s009.pdf]

$$\frac{dx_1}{dt} = -c_1 \cdot x_1 \cdot x_2 + c_2 \cdot x_3 - c_3 \cdot x_1$$
$$\frac{dx_2}{dt} = -c_1 \cdot x_1 \cdot x_2 + c_2 \cdot x_3 - c_3 \cdot x_2$$
$$\frac{dx_3}{dt} = c_1 \cdot x_1 \cdot x_2 - c_2 \cdot x_3 - c_3 \cdot x_3$$
