# Supplementary material for: Mathematical Model of the Firefly Luciferase Complementation Assay Reveals a Non-Linear Relationship between the Detected Luminescence and the Affinity of the Protein Pair Being Analyzed
Source: PLoS One. 2016 Feb 17;11(2):e0148256. doi: 10.1371/journal.pone.0148256 (PMC4757408; doi:10.1371/journal.pone.0148256)
Supplement: S2 ODE — These equations were obtained using knowledge of the enzymatic activity of NFLuc, as shown by the literature. For the purposes of publication, the numbering system was conserved between the full in vitro FLCA ODEs by removing the portions involving interaction with CFLuc. (PDF) [file pone.0148256.s010.pdf]

$$\frac{dx_1}{dt} = -c_9 \cdot x_1 \cdot x_6 + c_{10} \cdot x_{16} - c_7 \cdot x_1 \cdot x_3 + c_8 \cdot x_{15} - c_{26} \cdot x_1 \cdot x_{14} + c_{27} \cdot x_{19} - c_{28} \cdot x_1 \cdot x_{12} + c_{27} \cdot x_{20} - c_{15} \cdot x_1 \cdot x_9 + c_{16} \cdot x_{18}$$

$$\frac{dx_3}{dt} = -c_7 \cdot x_1 \cdot x_3 + c_8 \cdot x_{15} - c_7 \cdot x_{16} \cdot x_3 + c_8 \cdot x_{17}$$

$$\frac{dx_6}{dt} = -c_9 \cdot x_1 \cdot x_6 + c_{10} \cdot x_{16} - c_9 \cdot x_{15} \cdot x_6 + c_{10} \cdot x_{17}$$

$$\frac{dx_9}{dt} = -c_{17} \cdot x_1 \cdot x_9 + c_{18} \cdot x_{18}$$

$$\frac{dx_{12}}{dt} = -c_{28} \cdot x_1 \cdot x_{12} + c_{27} \cdot x_{20}$$

$$\frac{dx_{13}}{dt} = -x_{13} + c_{20} \cdot x_{18} \cdot (1 - c_{29})$$

$$\frac{dx_{14}}{dt} = -c_{26} \cdot x_1 \cdot x_{14} + c_{25} \cdot x_{19}$$

$$\frac{dx_{15}}{dt} = c_7 \cdot x_1 \cdot x_3 - c_8 \cdot x_{15} - c_9 \cdot x_{15} \cdot x_6 + c_{10} \cdot x_{17}$$

$$\frac{dx_{16}}{dt} = c_9 \cdot x_1 \cdot x_6 - c_{10} \cdot x_{16} - c_7 \cdot x_{16} \cdot x_3 + c_8 \cdot x_{17}$$

$$\frac{dx_{17}}{dt} = -c_{13} \cdot x_{17} + c_{14} \cdot x_{18} + c_9 \cdot x_{15} \cdot x_6 - c_{10} \cdot x_{17} + c_7 \cdot x_{16} \cdot x_3 - c_8 \cdot x_{17}$$

$$\frac{dx_{18}}{dt} = c_{13} \cdot x_{17} - c_{14} \cdot x_{18} - c_{20} \cdot x_{18} + c_{17} \cdot x_1 \cdot x_9 - c_{18} \cdot x_{18}$$

$$\frac{dx_{19}}{dt} = c_{20} \cdot (1 - c_{29}) \cdot x_{18} + c_{26} \cdot x_1 \cdot x_{14} - c_{25} \cdot x_{19}$$

$$\frac{dx_{20}}{dt} = c_{20} \cdot c_{29} \cdot x_{18} + c_{28} \cdot x_1 \cdot x_{12} - c_{27} \cdot x_{20}$$
