# Supplementary material for: Mathematical Model of the Firefly Luciferase Complementation Assay Reveals a Non-Linear Relationship between the Detected Luminescence and the Affinity of the Protein Pair Being Analyzed
Source: PLoS One. 2016 Feb 17;11(2):e0148256. doi: 10.1371/journal.pone.0148256 (PMC4757408; doi:10.1371/journal.pone.0148256)
Supplement: S3 ODE — These equations were added to the in vitro FLCA ODEs to model the IC-50 data obtained when adding nutlin-3 to NFLuc-p53 and CFLuc-mdm2. (PDF) [file pone.0148256.s011.pdf]

$$\frac{dx_{22}}{dt} = -c_{31} \cdot x_{22} \cdot x_{23} + c_{32} \cdot x_{24}$$
$$\frac{dx_{23}}{dt} = -c_{31} \cdot x_{22} \cdot x_{23} + c_{32} \cdot x_{24}$$
$$\frac{dx_{24}}{dt} = c_{31} \cdot x_{22} \cdot x_{23} - c_{32} \cdot x_{24}$$
