# Supplementary material for: Mathematical Model of the Firefly Luciferase Complementation Assay Reveals a Non-Linear Relationship between the Detected Luminescence and the Affinity of the Protein Pair Being Analyzed
Source: PLoS One. 2016 Feb 17;11(2):e0148256. doi: 10.1371/journal.pone.0148256 (PMC4757408; doi:10.1371/journal.pone.0148256)
Supplement: S4 ODE — Accordingly, only the interaction and catalysis of the NC complex is modeled here, with all references to free NFLuc or CFLuc removed. (PDF) [file pone.0148256.s012.pdf]

## S4 ODE

Here the *in vitro* FLCA ODEs were stripped down to be able to represent full-length luciferase. Accordingly, only the interaction and catalysis of the NC complex is modeled here, with all references to free NFLuc or CFLuc removed.

$$
\begin{aligned}
\frac{dx_2}{dt} &= -c_3 \cdot x_2 \cdot x_3 + c_4 \cdot x_4 - c_5 \cdot x_2 \cdot x_6 + c_6 \cdot x_5 - c_{23} \cdot x_2 \cdot x_{12} + c_{24} \cdot x_{11} - c_{21} \cdot x_2 \cdot x_{14} + c_{22} \cdot x_{10} \\
&\quad - c_{15} \cdot x_2 \cdot x_9 + c_{16} \cdot x_8 \\
\frac{dx_3}{dt} &= -c_3 \cdot x_2 \cdot x_3 + c_4 \cdot x_4 - c_3 \cdot x_5 \cdot x_3 + c_4 \cdot x_7 \\
\frac{dx_4}{dt} &= c_3 \cdot x_2 \cdot x_3 - c_4 \cdot x_4 - c_5 \cdot x_4 \cdot x_6 + c_6 \cdot x_7 \\
\frac{dx_5}{dt} &= -c_3 \cdot x_5 \cdot x_3 + c_4 \cdot x_7 + c_5 \cdot x_2 \cdot x_6 - c_6 \cdot x_5 \\
\frac{dx_6}{dt} &= -c_5 \cdot x_2 \cdot x_6 + c_6 \cdot x_5 - c_5 \cdot x_4 \cdot x_6 + c_6 \cdot x_7 \\
\frac{dx_7}{dt} &= c_3 \cdot x_5 \cdot x_3 - c_4 \cdot x_7 + c_5 \cdot x_4 \cdot x_6 - c_6 \cdot x_7 - c_{11} \cdot x_7 + c_{12} \cdot x_8 \\
\frac{dx_8}{dt} &= c_{11} \cdot x_7 - c_{12} \cdot x_8 - c_{19} \cdot x_8 + c_{15} \cdot x_2 \cdot x_9 - c_{16} \cdot x_8 \\
\frac{dx_9}{dt} &= -c_{15} \cdot x_2 \cdot x_9 + c_{16} \cdot x_8 \cdot x_{18} \\
\frac{dx_{10}}{dt} &= c_{19} \cdot x_8 \cdot (1 - c_{29}) + c_{21} \cdot x_2 \cdot x_{14} - c_{22} \cdot x_{10} \\
\frac{dx_{11}}{dt} &= c_{19} \cdot c_{29} \cdot x_8 + c_{23} \cdot x_2 \cdot x_{12} - c_{24} \cdot x_{11} \\
\frac{dx_{12}}{dt} &= -c_{23} \cdot x_2 \cdot x_{12} + c_{24} \cdot x_{11} \\
\frac{dx_{13}}{dt} &= c_{19} \cdot x_8 \cdot (1 - c_{29}) - x_{13} \\
\frac{dx_{14}}{dt} &= -c_{21} \cdot x_2 \cdot x_{14} + c_{22} \cdot x_{10}
\end{aligned}
\tag{1}
$$
