# Supplementary material for: Mathematical Model of the Firefly Luciferase Complementation Assay Reveals a Non-Linear Relationship between the Detected Luminescence and the Affinity of the Protein Pair Being Analyzed
Source: PLoS One. 2016 Feb 17;11(2):e0148256. doi: 10.1371/journal.pone.0148256 (PMC4757408; doi:10.1371/journal.pone.0148256)
Supplement: S1 Code — (PDF) [file pone.0148256.s013.pdf]

```
function main
%%%%%%%%%%%%%%%%%
%%NC parameters
%luciferin
c3=184;
c4=2.999999605697860e+03;

%ATP
c5=30;
c6=4.799990417951311e+03;

%adenylation rate
c11=5.005032362403402e+02;
c12=1.075165513854852e-02;

%intermediate
c15=7.771865248970217e+01;
c16=3.471656490390523e+00;

%kcat
c19=2.186046521866479e-01;

%L-oxy
c21=8.296207031450622e+00;
c22=6.132100294976869e-01;

%L-AMP
c23=5.000500240288718e+01;
c24=2.276804780286590e-05;
%%%%%%%%%%%%%%%%%
%%N domain parameters
%luciferin
c7=1.840621857623435e+02;
c8=5.049917207781118e+03;

%ATP
c9=2.994060619739656e+01;
c10=2.049501880555152e+04;

%adenylation rate
c13=5.496651143939792e-02;
c14=1.100207814311677e-02;

%intermediate
c17=7.771865248970217e+01;
c18=3.471656490390523e+00;

%kcat
c20=3.993565543918900e-07;

%L-oxy N
c25=8.296207031450622e+00;
c26=6.132100294976869e-01;

%L-AMP N
c27=5.000500240288718e+01;
c28=2.276804780286590e-05;
%%%%%%%%%%%%%%%%%
%%shared parameters
%p53-mdm2
c2=2;
c1=9.2;
kd=.212;
```

```

%dark reactions
c29=2.870910570336138e-01;
%%%%%%%%%%%%%%%%%
%degradation during incubation
%c30=.00136;%37 deg heat
c30=0;%no heat
%%%%%%%%%%%%%%%%%
%determine initial concentration
ini_n=.05;
%ini_n=.15;
%ini_n=.45;
ini_c=ini_n;
%incub_time=1;
incub_time=120;
Q=[ini_n;kd;c2;c30;incub_time;ini_c;];
[nconc]=plot_KDp(Q);
initN=nconc(1);
initC=nconc(2);
initNC=nconc(3);
%%%%%%%%%%%%%%%%%
%%DATA
%%%%long term
ydata = xlsread('50nm split.xlsx','B2:B1202');
tdata = xlsread('50nm split.xlsx','A2:A1202');

%%%short term
%tdata=xlsread('50nm 4s.xlsx','A4:A43');
%ydata=xlsread('50nm 4s.xlsx','B4:B43');
%ydata = xlsread('150nm 4s.xlsx','B4:B43');
%ydata = xlsread('450nm 4s.xlsx','B4:B43');
%%%%%%%%%%%%%%%%%
%solve ODEs
options = odeset('RelTol',1e-6);%,'MaxOrder','1','BDF','on');
Xo=[initN;initNC;75;0;0;10000;0;0;0;0;0;0;0;0;0;0;0;0;0;initC;];
%Xo=[0;ini_n;75;0;0;10000;0;0;0;0;0;0;0;0;0;0;0;0;0;];
tspan=tdata;
[t,X]=ode23s(@p53ode,tspan,Xo,options,c1,c2,c3,c4,c5,c6,c7,c8,c9,c10,c11,c12,c13,c14,c15,c16,c17,c18,c19,c20,...
    c21,c22,c23,c24,c25,c26,c27,c28,c29);
%%%%%%%%%%%%%%%%%
%plot
%% normalize data
%ynorm=ydata./max(ydata);
%simnorm=X(:,13)./max(X(:,13));
%plot(tdata,ynorm,'ok',t,simnorm,'r','LineWidth',2);
%% use gain
%ygain=3.041357255478377e+06.*X(:,13);  %50 nM long term
%ygain=5.3513e+06.*X(:,13);  %50 nM short term
%ygain=6.7415e+06.*X(:,13);  %150 nM
%ygain=6.5734e+06.*X(:,13);  %450 nM
%plot(tdata,ydata,'ok','LineWidth',2);
%hold on
%plot(t,ygain,'r','LineWidth',2);
%% plot options
set(gca,'FontSize',12,'FontName','Arial');
xlabel('Time (s)');ylabel('RLU');
%% plot individual portions of ODE
%subplot(4,3,1);plot(t,X(:,4),'m','LineWidth',2);title('nca'); subplot(4,3,2);plot(t,X(:,5),'m','LineWidth...
    ',2);title('cnl');
%subplot(4,3,3);plot(t,X(:,7),'m','LineWidth',2);title('ncla');  subplot(4,3,4);plot(t,X(:,8),'m','LineWidth...
    ',2);title('nci');
%subplot(4,3,5);plot(t,X(:,17),'m','LineWidth',2);title('nla');  subplot(4,3,6);plot(t,X(:,18),'m','LineWidth...
    ',2);title('ni');
%subplot(4,3,7);plot(t,X(:,5),'m','LineWidth',2);title('cna');  subplot(4,3,8);plot(t,X(:,5),'m','LineWidth...
    ',2);title('cnl');
%subplot(4,3,9);plot(t,X(:,10),'m','LineWidth',2);title('noxy'); subplot(4,3,10);plot(t,X(:,14),'m','...
```

```
    LineWidth',2);title('oxy');
%subplot(4,3,11); plot(t,X(:,13),'m','LineWidth',2);title('light');%subplot(4,3,12); plot(tdata,ydata);
return
%%%%%%%%%%%%%%%%%%%%%%%%%%%%%%%%%%%%
function init=plot_KDp(Q)        %function to solve initial conditions
options = odeset('RelTol',1e-6);
initN=Q(1);initC=Q(6);initNC=0;
kd=Q(2);cr=Q(3);cf=cr/kd;deg=Q(4);
Xo=[initN;initC;initNC;];
if Q(5) == 0  %no incubation
       init=[Q(1);Q(6);0;];  %return provided initial conditions
       return
end
tend=Q(5);
tspan=0:tend;
[t,X]=ode15s(@odeKD,tspan,Xo,options,cf,cr,deg);
%% plot initial conditions
%plot(t,X(:,1),t,X(:,3),'Linewidth',2);
%set(gca,'FontSize',12,'FontName','Arial');
%legend('[N]','[NC]');xlabel('Time (s)');ylabel('Concentration (uM)');
A=X(tend,1);
B=X(tend,3);
C=X(tend,2);
init=[A;B;C;];
return
%%%%%%%%%%%%%%%%%%%%%%%%%%%%%%%%%%%%
function[dx_dt]=odeKD(t,x,cf,cr,deg)        %%ODE for initial conditions
dx_dt(1)=-cf.*x(1).*x(2)+cr.*x(3)-deg.*x(1);
dx_dt(2)=-cf.*x(1).*x(2)+cr.*x(3)-deg.*x(2);
dx_dt(3)=cf.*x(1).*x(2)-cr.*x(3)-deg.*x(3);
dx_dt=dx_dt';
return
%%%%%%%%%%%%%%%%%%%%%%%%%%%%%%%%%%%%
function[dx_dt]=p53ode(t,x,c1,c2,c3,c4,c5,c6,c7,c8,c9,c10,c11,c12,c13,c14,c15,c16,c17,c18,c19,c20,c21,c22,c23...
    ,c24,c25,c26,c27,c28,c29)
%ODE
dx_dt(1)=-c1.*x(1).*x(21)+c2.*x(2)-c9.*x(1).*x(6)+c10.*x(16)-c7.*x(1).*x(3)+c8.*x(15)-c21.*x(1).*x(14)+c22.*x...
    (19)-c23.*x(1).*x(12)+c24.*x(20)-c15.*x(1).*x(9)+c16.*x(18);
dx_dt(2)=c1.*x(1).*x(21)-c2.*x(2)-c3.*x(2).*x(3)+c4.*x(4)-c5.*x(2).*x(6)+c6.*x(5)-c23.*x(2).*x(12)+c24.*x(11)...
    -c21.*x(2).*x(14)+c22.*x(10)-c15.*x(2).*x(9)+c16.*x(8);
dx_dt(3)=-c3.*x(2).*x(3)+c4.*x(4)-c3.*x(5).*x(3)+c4.*x(7)-c7.*x(1).*x(3)+c8.*x(15)-c7.*x(16).*x(3)+c8.*x(17);
dx_dt(4)=c3.*x(2).*x(3)-c4.*x(4)-c5.*x(4).*x(6)+c6.*x(7)+c1.*x(15).*x(21)-c2.*x(4);
dx_dt(5)=-c3.*x(5).*x(3)+c4.*x(7)+c5.*x(2).*x(6)-c6.*x(5)+c1.*x(16).*x(21)-c2.*x(5);
dx_dt(6)=-c5.*x(2).*x(6)+c6.*x(5)-c5.*x(4).*x(6)+c6.*x(7)-c9.*x(1).*x(6)+c10.*x(16)-c9.*x(15).*x(6)+c10.*x...
    (17);
dx_dt(7)=c3.*x(5).*x(3)-c4.*x(7)+c5.*x(4).*x(6)-c6.*x(7)+c1.*x(17).*x(21)-c2.*x(7)-c11.*x(7)+c12.*x(8);
dx_dt(8)=c11.*x(7)-c12.*x(8)+c1.*x(18).*x(21)-c2.*x(8)-c19.*x(8)+c15.*x(2).*x(9)-c16.*x(8);

dx_dt(9)=-c15.*x(2).*x(9)+c16.*x(8)-c17.*x(1).*x(9)+c18.*x(18);
dx_dt(10)=c19.*x(8).*(1-c29)+c1.*x(19).*x(21)-c2.*x(10)+c21.*x(2).*x(14)-c22.*x(10);
dx_dt(11)=c19.*c29.*x(8)+c1.*x(20).*x(21)-c2.*x(11)+c23.*x(2).*x(12)-c24.*x(11);
dx_dt(12)=-c23.*x(2).*x(12)+c24.*x(11)-c27.*x(1).*x(12)+c28.*x(20);
dx_dt(13)=c19.*x(8).*(1-c29)-x(13)+c20.*x(18).*(1-c29);
dx_dt(14)=-c21.*x(2).*x(14)+c22.*x(10)-c25.*x(1).*x(14)+c26.*x(19);
dx_dt(15)=c7.*x(1).*x(3)-c8.*x(15)-c9.*x(15).*x(6)+c10.*x(17)-c1.*x(15).*x(21)+c2.*x(4);
dx_dt(16)=c9.*x(1).*x(6)-c10.*x(16)-c7.*x(16).*x(3)+c8.*x(17)-c1.*x(16).*x(21)+c2.*x(5);
dx_dt(17)=-c13.*x(17)+c14.*x(18)+c9.*x(15).*x(6)-c10.*x(17)+c7.*x(16).*x(3)-c8.*x(17)-c1.*x(17).*x(21)+c2.*x...
    (7);
dx_dt(18)=c13.*x(17)-c14.*x(18)-c1.*x(18).*x(21)+c2.*x(8)-c20.*x(18)+c17.*x(1).*x(9)-c18.*x(18);

dx_dt(19)=c20.*(1-c29).*x(18)-c1.*x(19).*x(21)+c2.*x(10)+c25.*x(1).*x(14)-c26.*x(19);
dx_dt(20)=c20.*c29.*x(18)-c1.*x(20).*x(21)+c2.*x(11)+c27.*x(1).*x(12)-c28.*x(20);
dx_dt(21)=-c1.*x(21).*x(1)-c1.*x(21).*x(15)-c1.*x(21).*x(16)-c1.*x(21).*x(17)-c1.*x(21).*x(20)-c1.*x(21).*x...
    (19)+c2.*x(2)+c2.*x(4)+c2.*x(5)+c2.*x(11)+c2.*x(10)+c2.*x(7)-c1.*x(21).*x(18)+c2.*x(8);
dx_dt=dx_dt';
return
```
